# TNT Loss: A Technical and Nontechnical Generative Cooperative Energy Loss Detection System

**DOI:** 10.3390/s22187003

**Published:** 2022-09-15

**Authors:** Netzah Calamaro, Michael Levy, Ran Ben-Melech, Doron Shmilovitz

**Affiliations:** 1 Faculty of Electrical and Electronics Engineering, Tel Aviv University, Tel Aviv 6997801, Israel; 2 17 Lechy Street, Bnei Brak 5120026, Israel

**Keywords:** smart metering, smart grid, technical and nontechnical loss (TNT loss)

## Abstract

This paper describes an electricity technical/nontechnical loss detection method capable of loss type identification, classification, and location. Several technologies are implemented to obtain that goal: (i) an architecture of three generative cooperative AI modules and two additional non-cooperative AI modules for data knowledge sharing is proposed, (ii) new expert consumption-based knowledge of feature collaboration of the entire consumption data are embedded as features in an AI classification algorithm, and (iii) an anomaly pooling mechanism that enables one-to-one mapping of signatures to loss types is proposed. A major objective of the paper is an explanation of how an exact loss type to signature mapping is obtained simply and rapidly, (iv) the role of the reactive energy load profile for enhancing signatures for loss types is exemplified, (v) a mathematical demonstration of the quantitative relationship between the features space to algorithm performance is obtained generically for any algorithm, and (vi) a theory of “generative cooperative modules” for technical/nontechnical loss detection is located and mapped to the presented system. The system is shown to enable high-accuracy technical/nontechnical loss detection, especially differentiated from other grid anomalies that certainly exist in field conditions and are not tagged in the universal datasets. The “pooling” architecture algorithm identifies all other loss types, and a robotic process automation module obtains loss type localization. The system feeds from the entire smart metering data, not only the energy load profile. Other solutions, such as a stand-alone algorithm, have difficulty in obtaining low false positive in field conditions. The work is tested experimentally to demonstrate the matching of experiment and theory.

## 1. Introduction

Technical and nontechnical losses are referred to here as a collection of energy loss anomalies: (1) theft/fraud, (2) meter phase disconnect, (3) meter reversed-phase connect, (4) direct meter broken bridge between voltage plus and minus, (5) export energy at residential premises indicating losses for an arithmetic metering method, (6) meter bypass by some of the load, and (7) leakage to unknown loads. Some anomalies, (2)–(7), may be the result of non-fraud or fraud, and energy damage is the same and is remotely indistinguishable. This is simple. (2)–(6) are events reported by the smart meter, not only AI detected. In the current paper, the “technical losses” term is used for anomalies that are “not nontechnical losses”, as defined by (1)–(7). This includes: (i) preventive maintenance other than items (2)–(7) above. Information losses that resemble energy losses are not in this category of TNT loss. Failures in the smart metering data chain are caused by faulty components, (ii) cyberattack, and (iii) insertion of textual customer data factors such as the premises address and socioeconomic status. Customers may appear to have the same loss detection confidence level while excluding textual data. Inserting textual data into the learning space modifies the result. The introduction follows.

A full survey on fraud/non-fraud technologies appears in [1], both in the introduction and preliminary chapters. Next is a survey of concepts beyond [1]. The current paper is a continuation of paper [1]. To a major extent, it goes significantly beyond what was presented there. In brief, the previous work [1] mainly addressed AI algorithms processing electricity load profiles of 15 min to 1 h periods or processing other periodic energy consumption.

The previous work [1] obtained a holistic system using multiple smart metering data sources. The following brief lists what was obtained and what was not to better comprehend what is innovative in the work presented here. Previous work: (1) The system contained several AI modules but was not cooperative—rather separated. (2) There were four expert knowledge-based features identifying theft. The concept of the preprocessor feature generation module, named “semi supervised learning” rather than the self-generating feature CNN, was introduced. It was not purely original, but there are fewer works on semi supervised learning than on self-generating learning for TNT loss detection. (3) The system was more of a “theft + technical losses” vs. a non-theft classifying system. (4) Foundations of a mathematical theft detection theory were developed. The understanding is that in virtual feature space, theft reduces the dimensionality of “theft” clusters when features each contain separate information: if PCA space is 3D, then the theft is 2D. (5) Principal component analysis (PCA) as a means of theft/non-theft enhancement and simple classification handling was presented. (6) The last innovation in [1] was a general presentation of “robotic process automation” (RPA) technology and an algorithm for locating faults along the smart metering data chain: from meter to the demilitarized zone at the interface with energy suppliers. This was the first mention of RPA technology for TNT loss detection. Designs that were absent in the previous work [1] and are presented in the current work are as follows: (1) expert knowledge-based feature count is incremented to eleven: there were four features previously. Consumption expert knowledge is an analysis that combines the entire energy load profile data into a few parameters. (2) The three AI modules were not cooperative; rather, they were separate. There were fewer AI modules. (3) There was no closed loop of iterative learning. There were not two time scales at which the detection system acts: short-term and long-term; only a short-term scale existed. (4) The mathematical theory of TNT loss detection lacked two components: (4.1) quantification using computational complexity of the relationship between the feature space and algorithm performance. (4.2) The theory of “generative cooperative modules” for technical/nontechnical losses, simultaneous training, same data, and discriminator/generator game application, was absent. There is a significant difference between the GCN and separate AI modules. (5) The anomaly pooling mechanism, which is a mechanism that enables discrimination of many fault types using $O\left(N*logN\right)$ scenarios rather than the previous $O\left(N^{2}\right)$, was absent. Scenarios are mostly data augmented. Without pooling, it is impractical to discriminate between many fault types. (6) The fusion of multiple data into a single decision as regards to theft type was absent in [1] and is presented here. (7) The last technology innovated by the current paper is the presentation of the RPA algorithm for loss location. Here, more details are given regarding the technology by presenting the flow diagram. The bottlenecks shown in the previous work were that under field conditions (that defer from sterile fraud/non-fraud datasets), the non-fraud anomalies and information losses appeared generally, and not only in the presented work, to look similar to “features speaking” and very similar to technical losses. We defer to the survey, observing what’s been performed by others. A work presenting nontechnical loss detection in power grid is [2]. Another work presenting anomaly detection in power grid, and TNT losses are grid anomalies is [3]. Work [4] studied anomaly detection for smart meters in edge computing. It was demonstrated in [1] that if the faults are not discriminated from fraud, it will lead to a large false alarm rate. The problem statement of the current work is now defined. (i) Discriminate technical losses from non-technical losses and show exactly how it is performed. This was insufficiently presented in previous works. An algorithm is needed that does the job simply and intuitively in a theoretically provable and effective manner. (ii) identify exactly the TNT ‘loss types’ and learn from them with only a few validated scenarios, down to one/two scenarios per type. Let the AI architecture to be ready for new types. (iii) Localize the loss types along the smart metering data chain and along the distribution grid. The aim of this paper is to determine how to perform a. loss identification, b. loss type, and c. loss location. Since the publication of [1], the system has matured in six architectural enhancements, and the structure described here reflects these upgrades. The system is becoming the nation’s local utility electricity company’s TNT loss detection system. In paper [1], the foundations of a holistic technical + nontechnical (TNT) loss detection system were presented. The holistic system was shown to be multifold: (a) “sharing information with the smart metering system”. The smart meter event log file contains a variety of TNT events: (a. 1) magnetic tampering, (a. 2) reversed-phase, (a. 3) phase disconnect, (a. 4) open front panel, (a. 5) export energy at residential premises indicates a fraud suspect for arithmetic metering mode meters [5,6]. (b) The system is “holistic in the sense of embedding expert knowledge in a preprocessor to anomaly detection AI module, and not satisfactory with anomaly detection of energy load profile”; (b. 1) a mathematical foundation for electricity fraud is presented. A mathematical relationship between meter events and nontechnical losses detected by analyzing electricity load profiles is proposed. (c) “Mentioning at a glimpse that a TNT system may be extended to “fault detection” of an arbitrary count of anomaly types”. (d) Most importantly, “anomaly pooling: generate an ordered vector {0,1,0…,1} = {0 normal, 1 abnormal} that 1:1 uniquely maps according to our opinion, bound to testing, to fault/theft type is presented. The present paper may be regarded as a continuation of the development of the previous paper, not by a greater detail of each component, but by describing the additional architectural enhancements implemented to enable detection of any loss type at any location. Then, the paper demonstrates the detection of eleven exemplary fault types by the TNT system, all of which were verified as concrete faults. These statistics are close to a 95% true positive rate and less than a 5% false positive rate in real field conditions and not over a sterile dataset. To identify specific loss types and loss locations, over a wide set of loss types, the following innovations are proposed and implemented. (1) A set of three AI modules that function as “generative cooperative modules” are introduced. Initially, when there is a lack of tagged data, they are implemented as classical machine learning modules that require less data than deep learning. Eventually, when validated tagged data increase, they may be implemented as convolutional neural networks (CNNs). These networks did not exist in [1] as cooperative networks; in that work, they were separate modules. (2) The second innovation is the addition of new expert knowledge-based features, adding to the set of other expert-knowledge features described in [1], such as the insertion of reactive energy and spectral analysis. Each added expert-knowledge feature provides a new method for extracting new information from the same energy load profile input. (3) A mathematical background is presented. A mathematical algorithm’s analysis using computational complexity theory is introduced, and its ability to explain how expert knowledge-based features quantitatively improve the identification of various loss types is investigated. Can the quantitative effect of adding new features on the algorithm’s performance actually be measured? This paper shows what inserting new features does to the performance: the algorithm’s accuracy, the algorithm’s required training dataset, and the required training time. The aim is to obtain less training time, a smaller dataset, and higher accuracy. (4) Foundation: A mathematical theory of “generative cooperative networks” (GCN) explaining what is considered the physical behavior of the TNT loss system is indicated and mapped from “GCN for visual recognition” to TNT loss detection. (5) An identification of eleven loss types and loss localization within the smart metering data chain is demonstrated. Some loss types are information loss disguised as energy loss, and some are energy loss. Most importantly, a new “anomaly pooling” technology is explored: what happens if each expert knowledge-based feature is pulled to a binary indicator anomalous/normal. Then, the indicators are combined into an ordered vector $\left\{l_{1},l_{2},\ldots ,l_{n}\right\}$, called “anomaly pooling” here. Anomaly pooling is a technique for down-sampling. A lengthy vector has the ability to generate a large number of $2^{n}$ combinations. Mapping the pooling layer to {loss type, loss location} is studied. It is a method for identifying unlimited loss types. Mapping is not expected to be 1:1. For a large mapping vector, several vectors with single bit variation may be mapped to a single fault type. (6) The last technology is “loss location”. The smart meter is located at the premises. The anomalies originating at the premises all resemble what was demonstrated both experimentally and theoretically in the previous work on information loss and are demonstrated additionally in the current paper. There are information loss anomalies located somewhere between the smart meter and the last component along the smart metering data chain. Information loss and energy loss look very similar. Information loss is a data mismatch at the interface of the load profile data transfer between two smart metering modules due to software defects. It looks very similar to energy loss. This will cause a multitude of false positive alerts, and launching a team in the field is costly. To prevent this, the last layer is developed as the “loss location”. This is implemented using a technology named “robotic process automation” (RPA). Such a detection rate in loss detection, as is targeted here, deserves attention as to how it can be achieved based on the architecture. Expert knowledge-based features are enhanced. The criterion for selecting expert knowledge-based features is the ability of the feature “to collaborate the entire load profile data to a few parameters”. The expert features presented in [1] are enhanced from four to eight: (i) “spectral image” of the electricity load profile is added. Previous works implemented power spectral analysis, called “energy” spectral analysis here, (ii) the contribution of the reactive energy load profile when it exists is researched. It makes sense that it may contribute to the load’s technical loss detection; (iii) the reactive energy property is multiplied by all the other features because all other features are operating priory on the active load profile. The use of a “reactive energy load profile” for “technical and nontechnical loss detection” is one of the innovative presentations here, but it is not the first work on the subject. Analysis of reactive power was performed in [7] by de Souza et al. in 2020. Most smart meter types have an energy load profile rather than power; (iv) above these features, “meta-features” are now implemented using principal component analysis and correlation heat-maps (iv-1) and dimensionality reduction is known to enhance abnormalities. The 3D dimensionality reduction transformation principal component analysis (PCA) is used to generate a visual subspace per every expert knowledge-based feature; (iv-2) now added are PCA graphs of (1) “spectral” features (2) and “reactive” features, plus {all previously presented features} performed on the reactive energy load profile, (3) energetic distribution, (4) daily-hourly trends, (5) seasonal-hourly trends, and (6) spectral; (iv-3) a new meta feature, where all the generated features, approximately 256 features, times energy types {active, reactive}—denotes 512 statistical parameters; all are correlated using the “Pearson correlation heatmap “ for loss detection, generating a 2D carpet signature of the phenomena, the most collaborative image that can be generated. The “generative cooperative modules” implemented here are the motivation for the idea. There are multiple sources of data available for energy loss detection: (1) energy load profile, (2) meter event log file, (3) customer textual information, (4) “non-energy measurements based assertion rules”, (5) for “load location”, an additional source of data is available, the same {load profile, event log data}—reading from several stations along the data chain, and (6) the binary “anomaly pooling” vector, extends fault type identification beyond all those previously mentioned and covers all previous indications. When multiple sources are to be processed, then the solution is “tri-agent AI modules”—each processes a different group of data with the same time stamp. To collaborate the data, the suggested implementation is a game. Literature survey: Technical and nontechnical loss detection is a zero-sum game between the electricity utility company and the endpoint: presented in [8] by Cardenas, and a thorough review of the theft detection game theory works is found in [9]. What is saved by the company is “lost” by the endpoint. The work by Cardenas et al. focused on theft detection as a zero-sum game and used Nash’s equilibrium condition to detect some parameters that deferred between agent #1 “representing customer” winning or agent #2 winning (representing the company). There was a preliminary idea to represent each side by an agent and use a “generative adversarial network” (GAN) architecture [10], since GAN is a zero-sum game implementation and using GAN for theft detection, not for data augmentation, as presented in [11]. Two major problems with this idea are as follows: (1) there are no representative data available to the customer. Both the electric load profile, processed by all other TNT loss algorithms in the field, and the meter event log file are utility company representatives: two agents represent the company. GAN is used for TNT loss problems to generate augmented data for electricity theft [12], which is similar to the generator function. (2) The second and most important factor affecting not implementing GAN is that GAN consumes many more training scenarios than a single CNN. It may be comprehended intuitively from the fact that the generative network proposes false or noisy data. It takes the discriminator network a long time to train. In practice, this research group experienced a factor of 50 times more data. An alternative approach is tested is the “generative cooperative modules”. Not only do they share knowledge, but they also train simultaneously over the same data. That cuts the training effort: single networks training, single data. The theory of “generative cooperative modules” (GCM) is briefly presented and intensely referenced. The work by Xie et al. [13] is dedicated to generative cooperative networks for image processing: cooperative training of generator and discriminator, and the work [14] by Dai et al. for CNN generative cooperative networks. The current paper in its mathematical section contains two theories. One of them is a mathematical theory of “generative cooperative modules” for technical and nontechnical loss detection. This theory rewrites the variables of work [13], originally intended for image processing, to demonstrate that the theory describes the proposed “TNT loss detection” system well. Initially, the AI entities are classical machine learning modules; eventually, when tagged validated data increase, they may be replaced with “generative cooperative networks”. The proposed architecture is different from classical GCN in four fine-tuning issues that do not affect it being a GCN: (i) initially, neural networks are not used due to training cost and lack of validated data. Simple classifiers combined with expert knowledge-based features are shown to converge quickly. Initially, there are few validated scenarios. (ii) The training dataset is not exactly the same for the generator and discriminator. However, the time stamp is the same; therefore, a vector containing all data concatenated is the same for all modules, which is why it is correct to consider all cooperative modules receiving the same dataset. Each module is processing segments of these data. (iii) The training is not conducted in advance here but on-the-fly. Each new scenario is trained while the system is ongoing, functioning as TNT loss. (iv) Finally, the last difference is that there are two time frames: slow and real-time, working simultaneously. For real-time—each AI module works independently based on the validated loss types dataset. The system is explained thoroughly and graphically. To perform computations of training effort and accuracy and develop: (1) a computational formula of accuracy; (2) and a computational formula of training effort, one is required to discuss “information” as a quantity and information theory, there needs to be a quantitative measure about information. For some surveys, rule-based theft detection by Otuoze et al. [15], training networks faster by Mahabadi et al. [16], EU-28 benchmarking document [17], clustering-based analysis by Giannou [18], and feature semi-supervised learning for load identification [19] will be of use in the coming subject development. Many works on grid analytics use many datasets to train [1], and all operate under the comprehension that training a grid analytics algorithm, specifically TNT loss, is an empirical procedure that includes measurement rather than an assessment of the performance. Information is a quantity definition used in “algorithmic computational complexity” [20]. The less accurate an algorithm, the more information it contains, and the more scenarios are required to train it. The algorithm’s computational complexity has been developed, for example, by Avi Wigderson from Princeton [21] and Abel prize laureate 2021. There is a similar work in the line of thought to the proposed research of computational complexity works by Goldreich from HUJI, and Vadhan [22,23], also a work by Murawski et al. [24]. To compute information, one requires the information theory measure of “entropy”. A book by Barak and Arora [25] uses entropy for computational complexity. The work by A. Shashua et al. [26,27] from HUJI on deep learning networks and quantum entanglement again uses entropy to collaborate network behavior. Similarly, entropy is used to quantize information and not in quantum mechanical models of AI. The work by N. Tishby and N. Zaslavsky et al. [28] and Tishby and Bialek [29] from HUJI and MIT, the authors of “information bottleneck theory”, are considered by many as the best explanations with regard to how deep neural networks work. The work by G. Hinton from Toronto University and [30] and of Ghojogh et al. [31] on “deep belief Boltzmann machine networks” is also considered. The use of entropy in AI computation complexity is performed by a wide community of leading researchers. That computation is important in the context of the TNT context to explain how expert knowledge-based feature generation is extremely important to all three performance properties and to intensify the presented work to its rightful location.

The mathematical section includes, as well as the “information” and “entropy”, a background on the mathematics behind “generative cooperative networks” [13] and “generative adversary networks” [14]. The insertion of deterministic information networks and meter event log files into “TNT loss” detection is new. That information is not absolutely deterministic as is initially contemplated. Meter event log file events may be false. That information is suggestive rather than deterministic, which is why the module is named “generator”. The RPA is used in two AI modules as detailed later, generator—specifically, the submodule for loss location, and the probabilistic confidence level computation. There are at least a dozen recommended RPA platforms. For example, three cyber-approved ones at local electricity utility companies are mentioned here: Eggplant-software [32], UIpath [33], and Ranorex [34]. The selected platform here is Eggplant software. In addition to the planned theoretical issues, the architectural propositions and the new expert knowledge-based features come from the anomaly “pooling” technique of down-sampling the detection of features in feature maps. That proposed technology is simplifying and whether it is sufficient is investigated. If it is, then it makes loss classification simpler, each feature is modular and no requirement exists for full blown, high-order dimensional space, including all features except for small subspaces for each feature. Finally, with regard to the proposed TNT loss theory’s suitability for industrial premises, there are not only conventional grids but also second-generation power component loss identification systems, such as a loss-free resistor [35] and gyrator [36].

## 2. Materials and Methods

### 2.1. An Introduction to the System’s Architecture

After all that has been declared, the proposed system behaves exactly the same as the physics of GCN, as shown in the mathematical section during the long lifespan of the system. The proposed research considers that there are two agents representing the utility company: the first agent, “the discriminator”, processes (1) the energy load profile in expert knowledge-based generated features and (2) customer database textual data. The second agent, “the generator”, processes smart metering additional data sources: (3) the meter event log file, (4) expert knowledge assertion rules AI module, which is loss detection that human experts develop. For example, voltage≠0, current=0 indicates a disconnected phase, (5) which denotes the most important unknown faults implied by the energy load profile expert-knowledge pooling vector. Generating an anomaly pooling vector {0, 1, 0, …, 1} is the most important insert technology because this work demonstrates that this maps 1:1 or N:1 to fault type. The second importance is that the pooling tag expands the count of separable fault types up to $O(2^{N})$**.** The third importance of pooling is that it reduces the required training scenario count from $O\left(N^{2}\right)$ **to** $O\left(N*logN\right)$**,** while most of these are data-augmented for training acceleration. A previous work on rule-based theft detection is [15] by Otuoze et al. A difference from previous works is that here, “assertion rules” are one agent out of three cooperative agents. The “generator” generates the speculated {loss type}, and the “discriminator” discriminates whether that “loss type” is correct. The data presented by the “generator” is mainly not speculative, another fact in addition to “cooperative training” over the same dataset, that should shorten training. In addition to expert knowledge-based feature enhancement, the relationship of two intelligent proposed systems are described more in-depth architecturally as the “generator” and “discriminator”. The first is an artificial intelligence (AI) system that detects anomalies using an expert knowledge preprocessor. The primary AI system, the “generator”, is a robotic process automation (RPA) system that performs the following functions: (a) parse the meter’s events log file that is located at the data warehouse. The RPA system accesses smart metering modules, using the “remote desktop protocol” (RDP) to any unit under test (UUT) by visualizing the unit graphic user interface (GUI)—using OCR (optical character recognition). That module operates through the menus similar to the way in which a human operates; thus, it is named RPA. Parsing is required because the event log file includes approximately 100 events, and it is required to classify them into groups. (b) The second module named the “discriminator” performs a second objective in addition to “load profile expert knowledge-based features”. If there is an interface to customer textual data, then it is also inserted into the AI learning space. The third module, named “Module 3”, computes the probabilities as a function of the input provided by the meter. The “discriminator” teaches itself and the entire NT loss AI system to later identify the loss types based on their expert knowledge signatures, regardless of the primary deterministic meter event log event data. There is a fourth AI module, also an RPA, responsible for “loss allocation” along the smart metering data chain. Instead of speaking generally on “technical and nontechnical loss detection”, the entire system suggests a method (1) to identify, (2) classify, and (3) localize energy/information losses. This capability is demonstrated over eleven suspected loss types. Summarizing this paper, the focus is on several related objectives: (1) significantly enlarge the expert knowledge-based features presented in work [1]; (2) use the enhanced system for loss detection (loss/not loss), loss classification to loss type, and loss location. This is the most important objective of this paper. There are few works on generic methods for smart metering systems that are suitable for detecting any technical and nontechnical loss, identifying the root cause, and allocating the loss. The previous paper focused on fraud. This paper focuses on enhancing fraud detection and a set of eleven technical losses detected through a single system. Teaching AI the differentiation of many tagged loss types requires an enormous number of validated scenarios. Upon observing known datasets, there is no such known dataset. One of the objectives of this paper is to describe a realistic system that learns various loss types with a reasonable quantity of data. (3) Another objective of this paper is to continue laying a mathematical foundation for “technical and nontechnical loss detection” theory. Previously, it was demonstrated that it is no coincidence that TNT loss clusters drawn in PCA 3D space are one dimension reduced, meaning they are closer to the axes. The current mathematical foundation continues the theory development. The mathematical section of the paper includes two sub-objectives: (i) to present a computational work that demonstrates the role of the features space implemented by the expert knowledge-based features preprocessor on the algorithm performance through three parameters: (ii. 1) training dataset size: how many scenarios are required to train any architecture, (ii. 2) the algorithms accuracy upper limit, and (ii. 3) training dataset learning time. Theoretical work attempts to demonstrate that for two corner cases, obvious solutions are obtained, (ii. 4) this indicates that the “computational complexity theory of algorithm performance” is correct and may be used for algorithm design. The computation is shown to enable AI algorithm enhancement, specifically, TNT losses in phase transitions, from an exponential dependency of required “training effort” to a linear dependency. That it is fast training is shown. The second theoretical presentation is on the physical foundation of “generative cooperative modules” for TNT loss detection. (4) The next objective after laying a mathematical theoretical foundation is the presentation of eleven actual field-detected loss types and the demonstration of pooling technology for one-to-one mapping of loss type. (5) The next objective is to demonstrate the loss location along the smart metering data chain.

### 2.2. A Variable Algorithm Suitable for Various Local Dataset Sizes

There is an important reason why expert knowledge-based feature generation preprocessor prior is embedded in an anomaly detection core. At the beginning of the fraud system operation, there are large universal datasets [37,38,39] without “technical/nontechnical loss detection” tagging and without differentiation from other grid anomaly types: energy losses and information losses, such as metering equipment maintenance issues. Therefore, performing further learning on a local dataset is required. At the beginning of operating such a system, there are not too many verified frauds and no verified technical losses according to loss types. Therefore, a system that can learn quickly must be provided. How to cause deep networks to generate consumption of expert knowledge features independently remains a subject for future research. Until then, a preprocessor is considered as needed. (1) The proposed algorithm should enable a “technical/nontechnical loss detection” signature that is not dependent on a non-loss status reference from that same customer or from that customer profile. The research investigates whether there is such a thing as (any) loss signature and the loss type signature. (2) The distinguishability between various loss types is very important, and here it is shown that in all loss types, the features appear to be too similar. However, pooling each feature into anomalous/normal indicators and ordering the indicators inside a string vector potentially maps the 1:1 loss types to that $\left\{0100110\right\}\;$ vector. There are eight “technical/nontechnical loss detection” datasets universally, and four are surveyed in [1]. They do not provide tagging for anomalies that appear to be fraud, feature speaking, as the work in [1] demonstrated both theoretically and empirically, and the current paper continues to demonstrate empirically. The local utility company verified energy thefts and verified nontechnical losses are initially usually rare. There is no certainty of distinguishability from other anomalies at training performed only on international theft/no-theft datasets. Likewise, many field-tested algorithms show that the “true positive” rate will occasionally be inferior to the rate reported in other works, and the “false positive” rate will occasionally be higher. (3) Due to items (1)–(3) above, and six technological enhancements (1)–(6) in the introduction (expert features, anomaly pooling, mathematical theory, etc.)—the algorithm proposed in the current paper is shown to be holistic in physical implementation: (a) sampling across smart metering system modules, from meter to a data warehouse, (b) computing “certainty level probabilities” from system modules unreported by other works, and (c) considering a nested algorithm, GCN is an algorithm within an algorithm that improves learning with detected loss types. Considering generative cooperative modules that share data and learning data, (d) this work investigates three lifetime algorithm stages: (d-1) infancy: <100 verified frauds in dataset, (d-2) maturity: <1000 verified frauds in a local dataset, and (d-3) steady-state: >1000 verified frauds and other grid anomalies than theft over local validated technical/nontechnical losses dataset. These stages are expected to be affected according to the proposed research: (a) a different classifier algorithm is optimized to the best per range, (b) gradually, various loss types may be tagged in the local dataset, and the “technical/nontechnical loss detection” algorithm is shown to be capable of separating all of the loss types. That is relevant twofold, both for envelope enlargement but also because if the scenarios are untagged, there is no certainty that only fraud/non-fraud cases exist in a dataset. (4) Finally, an algorithm “with sockets” is desired for future enhancement. When additional information is available, it will be used, and the TNT loss system will function efficiently, even if the additional smart metering information, such as the meter TNT loss events log file, is not present. For example, in research, customer textual data should be included in a learning space: (i) address, (ii) socioeconomic implied status, (iii) family name (relatedness to theft family members), (iv) and customer type, at a relatively easy embedding. If an interface to a customer information database is absent, then learning is purely non-textual. Another future example is enabling future usage of a “technical/nontechnical loss detection” algorithm “at a distribution transformer” for remote detection of the same TNT losses detected at a single customer out of 100 customers. This will be a significant modification. The technical/nontechnical loss detected on a single premise is ~33–66% of the consumption. The technical/nontechnical loss of a single customer detected at a distribution transformer out of 100 customers is ~0.5%. How can this be enabled simply? In addition to designing an algorithm with a simple fallback solution, it must be designed to enable modularity.

### 2.3. A Holistic Multi Loss Type—An Algorithm System Instead of an Algorithm: Generative Cooperative Modules

**Figure 1:**
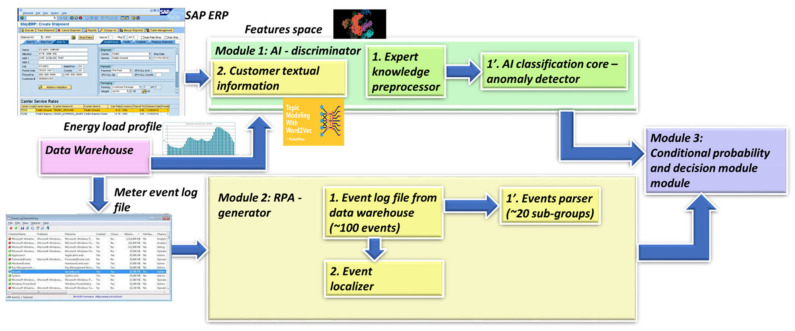
Inner algorithm flow diagram of the proposed algorithm—a holistic approach designed to reduce false positives and maximize true positives. GCN components are marked.

This section continues the uncompleted explanation of the TNT loss architecture from the work in [1]. It sheds light on the generative cooperative modules architecture. The focus of the previous paper was mainly on fraud/non-fraud. As previously noted, the system defined in [1] has matured. The multi-agent ingredient is a “tri-generative cooperative modules” structure, a new structure to the design, and turning all the submodules into “AI modules” is also new. This paper’s focus is on the following. (a) The expert knowledge enhancement is considerable compared to the previous work. From four to eight expert knowledge features were presented in [1], (b) this expert knowledge improves technical/nontechnical loss determination. It is intended primarily to enable the TNT system to act as a real technical/nontechnical loss: (i) detection, (ii) identification of loss type, and (iii) loss location. (c) The research does not satisfy expert knowledge enhancement compared to the previous work. This does not accomplish the goal of fluent technical/nontechnical loss detection, identification and location. To accomplish that structure, such that in the long run, through iterative training the proposed structure gradually behaves in the same way as “generative cooperative modules”, [13,14] (GCM) or “generative cooperative networks” are employed when there is a sufficient dataset. These are named “modules” because the initial architecture and training cooperate with AI modules, rather than networks, which enables the usage of fewer validated examples to proceed. Three intelligent module structures collaborate: (1) the first module, the “generator” AI module or “Module 2” in Figure 1, (i) receives meter generated events processed by the AI; (ii) uses expert knowledge to generate additional assertion rules for validation based on processing other non-energy load profile data from the smart meter, (iii) determines the second most important technology after pooling, “unknown and new failures generation” (marked NEW_FL). The module uses the signatures generated from the electricity load profile by Module 1.1 “expert knowledge preprocessor”. Applying the pooling mechanism described in Section 2.8 below, NEW_FL generates a binary vector {0,1,0, 0, …,1} of each signature, where normal = 0 and abnormal = 1. That signature vector is mapped to an $unknown\_failure\left[index\;i\right]$. The failure location module locates the failure. If the location is a meter, then a team is sent to the field. After a single iteration, the unknown failure is tagged with a known root cause and enters the hash to map known failures. In addition to the pooling mechanism and expert knowledge-based signatures, NEW_FL is the most important implementation. Meter events and assertion rules enable N failure types per *N* events. NEW_FL enables $2^{N}$ events for *N* signatures. $unknown\_failure\left[index\;i\right]\;$ with only a single digit difference from a tagged (known) failure is indicated as a suspected tagged failure. (2) The second module, the “discriminator” AI module or “Module 1” receives: (i) energy load profile data (ii) provided there is an interface to the billing system module that receives customer textual information: (a) address, (b) family name, (c) socioeconomic level derived from consumption profile, and (d) customer or contract type. (3) The third module, loss locator, is marked as “2. Event localizer” at “Module 2” in Figure 1. This module is implemented as a robotic process automation (RPA) system that allocates failures along the smart metering data chain, including at the premises. Figure 1 shows a holistic approach toward technical/nontechnical loss detection that is based on the field experience of many grid failures that appear to resemble fraud and AI signature speaking. The proposed algorithm is holistic in the following sense. In addition to these collaborating AI modules that are (1) sharing knowledge to construct a more perfected image and (2) sharing simultaneous training, there is a fourth non-collaborating module, which is (4) the decision-making module marked as “Module 3” in Figure 1. That module proposes a probability estimation of loss and loss type. Referring to Figure 1 marked modules: (1) “Module 1” in Figure 1 is the “discriminator” an AI module, the data warehouse, is the last module along the smart metering data chain, prior to propagating to the billing system (SAP ERP here) or to the network information system (NIS). Later, an AI “classification core” is implemented consisting of various technical/nontechnical loss types. (1.2) “Module 1” also receives non-mandatory existence-based “customer textual data” from the SAP ERP billing system. That data may also be inserted into the learning space by using the word2vec library [40,41,42], which turns words into.

**Figure 2:**
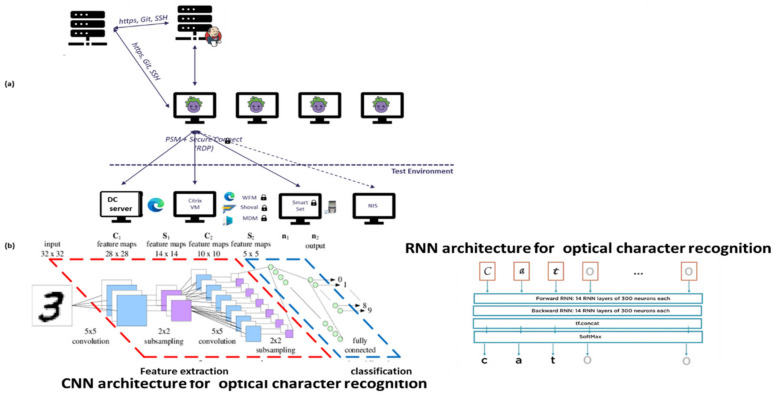
(**a**) How the “robotic process automation” is implemented on the smart metering system—schematic implementation using four “Eggplant software” licenses and accessing various components of the smart metering system. (**b**) OCR implementation using a convolutional neural network-left or recurrent neural network (LSTM) right. The OCR network is used by Eggplant for RPA implementation.

**Figure 3:**
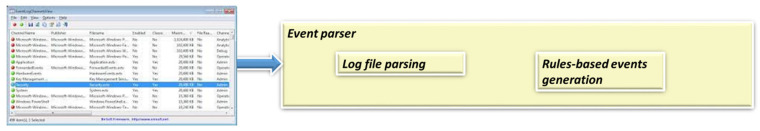
Zoom-in of events parser Modules 1 and 1′.

Numerical vectors. The “discriminator” module cannot initially identify all loss types. There are no international datasets with tagged verified technical losses, and initially, there are few verified tagged technical/nontechnical loss cases. Therefore, the information from “Module 2” shows that the generator is suggestive and not deterministic. By complementary information sharing from “Module 2”, the “generator”, Module 2-2: event localizer (a submodule of Module 2) localizes the failure location by comparing data between every two consecutive data stations along the smart metering data chain. It verifies that prior to dispatching a team to the field, loss is possible in the field, and if not, it assesses where it is located. These are cooperative modules. “Module 1”: the “discriminator” generates expert knowledge-based features over the electricity load profile. Module 3 performs the failure location based on a comparative analysis of the load profile, data model and energetic load profile between two successive chains along the smart metering data chain. These three modules are cooperative. The following is a stand-alone module. Module 4, a decision-making module, is the probability computation of the loss type and its confidence level. The cooperative modules are “generative cooperative modules” (GCM/GCN), a graphical structure similar to “generative adversary networks” but with totally different game rules: cooperative work and, here, cooperative training rather than adversarial. GCM/GCN is a win-win game striving to reach consensus. Finally, the architecture enables the cooperative networks to improve and become more accurate. (i) The “generator” generates (i. 1) loss type events and (i. 2) anomaly pooling vectors. The events are of deterministic origin from the smart meter, a fact that may accelerate learning but are speculative. For some meter model types, the sensor is incorrect. (ii) The “discriminator” provides anomalous/normal feedback about these events in the form of an expert knowledge-based feature that enables clustering of anomalous/normal. In the full-order dimensional space including all features, it is possible to identify and classify all the loss types. A technology named the “pooling layer” enables us to handle each feature as a stand-alone. (iii) The loss-type localizer locates the loss along the smart metering data chain. Cooperative training has a much longer cycle than independent training, a cycle of at least several iterations until the accumulation of enough tagged verified loss types has accumulated, such as to retrain the AI modules, which are now acting as a single system. Figure 2 shows the RPA operation. The long cycle duration may be accelerated; its duration is dependent on the availability of a synthetic “data augmentation” system that is currently implemented primitively. A formula will be developed as to the training effort and how this is affected by the Module 1 “expert knowledge-based generated features” over the load profile. In that dual life cycle, the GCN structure is somewhat different from a conventional GCN, it does not wait for the entire training effort to finalize. That is a significant difference. The GCN cycle: shall be shown to be the same as the computer vision GCN. System AI Module 2-2 is the “loss localizer”, and Module 2-1 is the “event log parser”. Initially, in the algorithm’s deployment infancy stage, the dataset is small and includes only validated thefts. The TNT loss detection and “loss localization” tasks are performed in the presented work by a technology used for TNT loss named “robotic process automation” (AI) software. The principle of an RPA, as described in Figure 2, is performed outside the systems under test (EUTs) through privileged access, the same way a human operator would access these systems, through a graphic user interface and menus and through visual recognition. The “event log parser” receives as input the smart meter event log file from the data warehouse. It performs several objectives: (2.1) “parse the event log file”, which may contain up to ~100 event types. Parsing means processing the meter event data into twenty event groups and extracting the information object from the textual line. Even as Module 1 retrains and is capable of diagnosing failure types, Module 2 is not turned off. It does not require considerable computational effort and enables new type detection. Gradually, when verified technical loss failures are collected into the local complementary dataset and tagged, the technical/nontechnical loss detection algorithm “discriminator” learns how to identify them using AI. (2) Module 2: (2.1) includes robotic process automation (RPA) that reads specific meter events that have arrived at the data warehouse. The data warehouse is intended for all grid analytics. Later on, Figure 1 is redrawn to demonstrate that this is truly a GCN-like architecture. Observing Figure 1, the parser includes two subcomponents: (i) Module 1′, a parser for smart meter events, and (ii) Module 1.2, for rule-based events: these are testing rules formulated by the TNT expert knowledge personnel, operated also over non energy load-profile data, to enable TNT loss detection. Not every loss detection is AI, and it is unwise not to use expert experience to embed it into the mechanized system. Two examples: (a) “if measured voltage v≠0, and measured current i=0, then that is a secondary method to deduce “phase disconnect suspicion”, secondary to the “disconnected phase event”; (b) “for an arithmetic metering method [5] meter, if $Energy_{Active,\;export}\neq 0$ for a residential customer”, then that is a suspicion of reversed-phase connect. (2.2) The second component of Module 2 is the “events localizer”. Its functionality is to localize the loss types along the smart metering data chain. To localize loss types, it must have access to load profile data at five points along the smart metering chain. This submodule functionality is described in Section 2.5 and Figure 2. The RPA was not implemented using UIpath, as reported in [1], but using Eggplant software [32] accessing through the RDP protocol to the smart metering modules. The coding effort at Module 2 in Figure 1 is at the “event localizer” using RPA, and not at the OCR networks computer vision AI—this is ready-made licensed software. Figure 3 below shows how it is embedded in the smart metering system, maintaining end-to-end automation of verification processes yet being cyber protected. The licenses identify the unit under test (UUT) graphical user interface using optical character recognition (OCR) AI, Figure 2a. To access all the UUTs of a smart metering system, the RPA resides on a separate server and not “inside” the smart metering system. Had it been installed inside the MDM, for example, even opening the ports between meter data management (MDM) and workforce management (WFM), would not have made it capable of operating the WFM. The preferred method is from the GUI. The RPA accesses the “meter data management”, the “workforce management” systems, the billing system “SAP ERP” and the “network information system” (NIS). Accessing using OCR denotes using a password and username, exactly as a human user accesses the EUT having privileged access using the remote desktop protocol (RDP). An RPA is just another “human user”. The neural network is at the OCR, not at the “Module 2” implementation, as previously shown in Figure 1, which is the classical software implementation. Figure 2b is a CNN implementation of the OCR. Off-the-shelf OCR software, such as OCRopus, is designed similarly to the Figure 2b recurrent neural network or combined CNN-RNN [43]. To determine the “graphical user interface” (GUI), other AI structures are required [44]. Figure 3 zooms in on submodules 1.1 in Figure 1. Events may be taken directly from the meter event-log file and are enhanced with expert knowledge rule-based events. The events are “suspicions” until they become deterministic. The explanation of statistical computation and transition to deterministic is explained in the probabilistic computation section and the retraining cycles section. A few words about the selection of the RPA architecture are as follows: (i) the first RPA implementation here, as simple as it sounds, is very sophisticated, and much experience has been gained for a TNT loss system; (ii) had the software been installed at the MDM, it would not have succeeded in performing end-to-end system operation, accounting for cyber systems. The difference between the two platforms is as follows: UFT is “hanging with hooks” to GUI code objects and communicating via ports, and Eggplant identifies optical images. The algorithm is complemented here, as shown in Section 2.6 below. If in Figure 1 Module 1.2. “customer information” is inserted into the learning space through the application of “word2vec” models for the representation of textual data into the vector space, then the algorithm fraud estimation accuracy increases. Information is inserted in the form of four attributes: (i) customer family name, (ii) address, (iii) implied socioeconomic status, and (iv) customer type. “Word to vector” is based on a pioneering work by PhD Tomas Mikolov in natural language processing (NLP) [40,41,42]. That method is supported by the Python library “word2vec” and enables usage by researchers who are initially non-expert NLP researchers. Taking two customers, one from a wealthy socioeconomic residential area and one from a poor socioeconomic residential background, with a high “technical/nontechnical loss detection” neighborhood rate, or two people belonging to the same family and living next to one another, one is verified fraud and the other is suspected—textual data modifies the probabilities. (3) Module 2.5, the fifth module in the algorithm, using the same TNT loss algorithm, enables the identification of super-consumption [1] and is not drawn here. Conventional energy fraud is sub-consumption. Super consumption detection enables the identification of either a third-party consumer using energy paid by another consumer or the identification of hidden premises in favor of maintaining public security work. Most importantly, super-consumption exists in the field and initially resembles fraud, in terms of signature and features.

### 2.4. Module 4: Decision Making, Probability Computation Prior to Sending a Technical/Nontechnical Loss Detection Team to the Field

**Figure 4:**
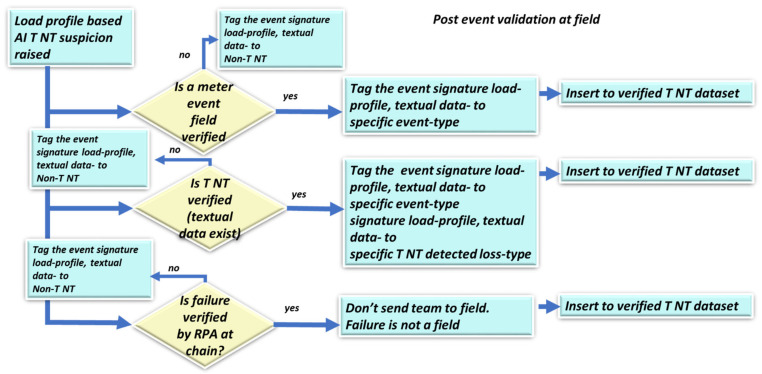
Algorithm for computing the probability of TNT loss suspicion in the field pre (left) and post (right) event validation. This is the feedback loop at the GCN structure in Figure 5.

This section complements the probability section of work [1]. This chapter is an evolution of that paper and recognizes that there is more than the scenario of “data mismatch along the smart metering data chain”. This section addresses (a) the method by which “events generated by Module 2 affect the confidence level (probability) of TNT losses” and loss type determination. Assume that an event is triggered by Module 2 either by the meter event log file or by assertion-based events; (b) the method for considering the addition of “customer textual data”, the probability that this is fraud, (1) the probability that this is a specific type of technical loss (2) or any technical loss, or (3) “either a technical or nontechnical loss”, and (c) the method applied when there is a suspicion of mismatch along the data chain, i.e., information loss and not energy loss, as handled in Section 2.5 below. Figure 4 outlines the algorithm for the probability distribution computation.

Observing Module (3) in Figure 1 of the energy “technical/nontechnical loss detection” system is the final and most important step in a holistic approach. Figure 4 shows the algorithms handling three types of scenarios; the left is prior to knowledge validation in the field, and the right is post knowledge validation in the field. The following are use cases where the decision is to send a team to the field for inspection. Case 1: “one or more meter events or assertion rules were raised”. Smart meters have approximately five events registered at the meter and from there to the fault manager at the “meter data management” MDM center and to the data warehouse where the TNT system has access. In any of these events, the policy is to raise the TNT probability or decision level to 1 indicating “send team to the field”:

(1) $$\begin{array}{l} \begin{array}{cc} \mathrm{se}t:dec(x_{i})=1 & p(\overset{N}{\underset{j=1}{\cup }}x_{j} \end{array})=1\\ set:dec_{T\_NT}(\vec{x}=loss)=1 \end{array}$$

where

$x_{i}$ denotes a specific meter event or assertion rule set at Module 1, the “generator”
$\cup_{j=1}^{1}x_{j}$ denotes any of the meter events or assertion rules is set
dec=1 denotes a decision to send the team to the field for inspection
$\vec{x}$ = loss denotes the TNT loss event
$d_{T\_NT}$ denotes the probability decision that it is a TNT loss

In the event that this is a false alarm for a specific meter model type, for the successive events this is marked as a “possible false event”; if there is a problem with the specific event sensor at that model type for some of the meters, it must also be replaced in the field.

Case 2: “there is customer textual data and based on experience (verified TNT loss dataset) it has probability $p\left(A\right)$ of being a TNT loss”. For example, there is a town named “WW”. Data are inserted using word2vec into the learning space using verified TNT cases. If $p\left(A\right)\;$ is higher than the threshold, then a team is sent to the field; otherwise, no team is sent.

Case 3: loss localization occurs when “any suspected TNT based on the load profile and textual data is verified as a failure along the smart metering data chain using the Module 2 localizer submodule by the Eggplant-software RPA”. The definition of localizer functionality is shown in Section 2.6 Figure 7. Upon receiving an alert from any of these events in cases 1 and 2 above, it is verified that it is not along the smart metering data chain. If verified to be in the data chain, then it is a fully localized data mismatch and not in the field. Otherwise, it is highly suspected to be TNT, and it is recommended to send a team to the field. Mathematically formulating that scenario immediately increases the “technical/nontechnical loss detection” probability to close to one based on the following conditional probability model:

(2) $$\begin{array}{l} p({\vec{x}}_{anomaly})=\sum_{n}p({\vec{x}}_{anomaly}|y_{n})p(y_{n}),\begin{array}{cc} & p(\vec{x}|\vec{y})=p(\vec{x}\cap \vec{y})/p(\vec{y}) \end{array}\\ \vec{y}=\overset{N}{\underset{i=1}{\cap }}not(y_{i})\begin{array}{cc} & \vec{y}=\left(y_{1},y_{2},\ldots ,y_{6}\right) \end{array} \end{array}$$

where:

$\vec{y}$—a logical operator and not being an event type from the following types:
$y_{1}$—“not a data mismatch anomaly” ∩
$y_{2}$—“not a preventive maintenance anomaly” (part of technical losses) ∩ ”
$y_{3}$—“not a cyberattack anomaly” ∩ customer information: $y_{4}-$“customer is not from high socioeconomic status” ∩ $y_{5}-$“customer is not abroad” ∩ $y_{6}-$“customer is not from town with a low fraud rate” ∩ $y_{7}-$“not super-consumption” ∩
$y_{N}-$“events from the smart meter included”—magnetic tampering and front-panel opening.
where the count of no events is from the total anomaly count and not from the entire specific customer count.
$\vec{x}$—event in which a customer with a “specific TNT loss type signature” from ∪ groups (i), i = *1,2..., N*.

The top left formula in Equation (2) states that an anomaly event is spanned by the scenarios described in (1)–(N) above and is not only a “technical/nontechnical loss detection” scenario. The top right formula states that a sterile dataset without tagging the non-fraud anomalies assumes that the anomaly is not one of the six scenarios (1)–(N) above. This means that in field conditions, algorithms that are not trained to separate these scenarios, at least “information loss” from “energy loss”, then the actual true positive rate will be lower than the reported rate over a non-tagged dataset. With regard to these anomalies, if not handled, the false positive rate is high.

### 2.5. A Holistic Technical/Nontechnical Loss Detection System—An Ever-Learning Algorithm—GCN-like Architecture

The discussion continues with respect to the development of an architecture that can identify and classify electric anomaly types in [1], with the reasonable presentation of previous material when needed. It is shown here that this is a “generative cooperative network” GCN-like design.

Theorem 1. “The GCN-like design”. The implemented design in Figure 1: (1) is equivalent to “reinforcement learning” and “generative cooperative networks (GCNs)”. These are collaborative modules rather than adversarial, and the “generator” is deterministic in nature but speculative according to model type. (2) The training cost is reasonable due to the difference between the GAN and GCN natures. In “generative cooperative modules”, to be more precise, there are initially no neural networks, only two AI machine learning modules. However, they are collaborative, similar to GCN networks. It is shown here that (i) a reinforcement learning structure is created and (ii) it is identical in operation to the “generative cooperative networks (GCN)” high-level diagram. This means that the collaborative modules replace the GAN rival “adversarial” competing networks.

**Proof.**  Let the input vector $\vec{X}$ consist of (a) the mandatory component of the smart meter energy load profile. $\vec{X}$ —consists of one to four channels for four energy quadrants, read by the “TNT” system’s “Module 1”. Different meters have different channel counts.

(3) $$\vec{x}=(active_{import},active_{\exp ort},reactive_{import},reactive_{\exp ort})$$

**Figure 5:**
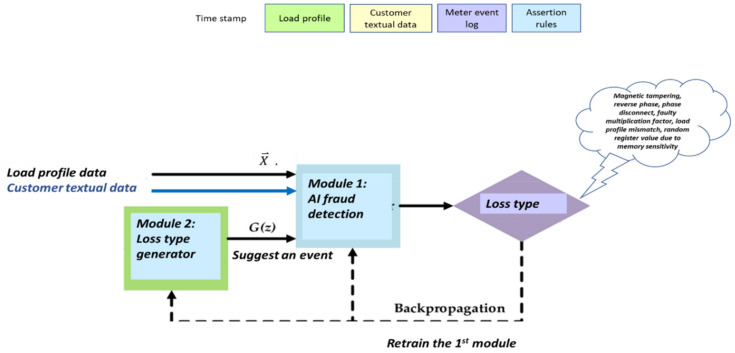
High-level schematic of the TNT loss detection system—it is the same as the generative adversary modules scheme but only when the retraining module is considered.

A second optional component serving as input to the system in Figure 5 below is customer textual data, received from the billing system. Such data includes customer address, customer family name and its relationship to nearby verified frauds, and customer socioeconomic status induced from electricity consumption. These data are converted from textual into numerical vector data through word2vec technology:

(4) $$\vec{y}=(customer_{name},socio\_economic,correlation_{verified\_frauds})$$

“Discriminator” module. Let “Module1” be the “AI technical nontechnical (TNT) loss detection system”. Figure 5 top shows that the same data train all modules’ concepts, “the same time stamp and each module selects the desired segment”. Training happens simultaneously, with the same scenario and the same data. The latter is crucial to understanding why these are cooperative modules. That module is constructed of two submodules: (i) an “expert knowledge preprocessor” that is detailed further here that includes eight expert knowledge features with unlimited growth potential; (ii) an optional “word2vec” based module that inserts two customer data parameters into a learning space: (ii. 1) the customer socioeconomic status that may be computed from the consumption profile and (ii. 2) “genealogy”—the customer family name indicating the familial relationship to another known/suspected fraud customer. “Generator” module; let Module2 be a secondary system that is “robotic process automation (RPA)”-based. It is constructed of the two elements shown in Figure 5: an (i) “event log parser” and (ii) an “event localization submodule”. These submodules are described later. “Real/Fake” or “loss type” determination submodule. Let the AI classifier implementing the Figure 5 “loss type” determination be the “real/fake” GAN module. It determines “fraud/no fraud” in the narrow sense and “fault or no-fault and fault type determination” in the broad sense. Let the “retraining algorithm” described in Figure 5 be the “backpropagation feedback loop”, which is a component of the GAN architecture. After the “discriminator releases an alert” and the “loss type” module recommends sending a fraud-detection qualified person to the field, the verified fault types from field tests are inserted into the dataset, and the “Module 1” + “loss type” is retrained over the dataset. Theoretically, by storing the pre -trained status, these two modules may retrain on the added examples.

Let $G\left(z\right)$ the “generator function from GAN architecture”, be constructed from a mandatory suggested “event type” from the meter, and optional suggested $\left\{family\;name,\;socio-economic,\;family\;relationship\right\}$ data.

**Figure 6:**
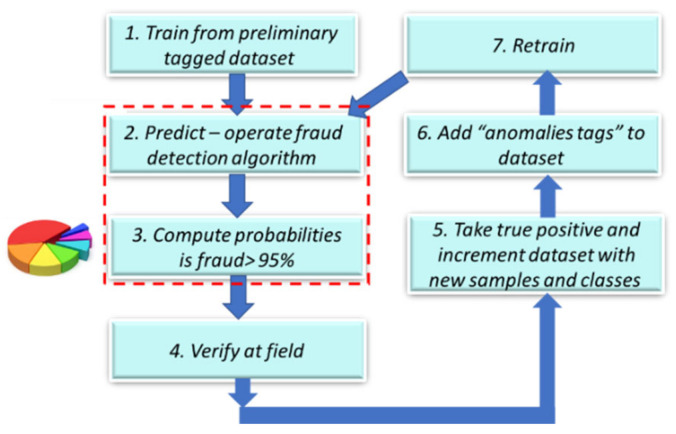
Iterative outer algorithm flow diagram. The dashed square marks the modules in Figure 1. The algorithm is an implementation of the feedback loop in Figure 5.

Let the “generator” suggest an event type $G\left(z\right)$ to the “discriminator”. That suggestion is performed by the conditional probability computation module described in Figure 6. Backpropagation is not a stochastic gradient descent but is a gradient, as shown later. Subsequently, in addition to the neural network, yes/no implementation of the “discriminator” and “generator”, the described system is a GCN because Figure 6 describes both the GAN and GCN presented architectures. □.

**Proof.**  Part 2: shorter training time than the GAN. The following may be formulated mathematically. (2.1) The GCN is both cooperative information and “simultaneous training” over the same dataset. GCNs may alternatively be without cooperative training [14]. The epoch count is simultaneous to both modules, which reduces the training effort, scenario count, and training time. Intuitively, cooperation leads to faster agreement, and an adversary leads to better readiness for complicated scenarios. (2.2) Module 2 is deterministic in nature, although it is speculative in the sense that the sensor may be wrong. The “generator” being deterministic reduces the training effort, provided it is correct most of the time. The issue is that if the information is more deterministic and less speculative, training accelerates because there is less uncertainty. In the mathematical section, the proof is presented that less certain systems require higher training effort and vice versa. (2.3) The pooling technology presented here considerably shortens the training time, which is discussed in the “pooling section”. The proof is in splitting a multidimensional space of size *N* into segments of size *n* and simultaneously processing each segment that shrinks the training effort computationally as $log\left(N\right)$, compared to log $\left(N/n\right)=\log\left(N\right)-\log\left(n\right),$ because training in the worst case is collaborative multidimensional and therefore exponential. □

Experimental evidence: for example, electricity load forecasting CNN/LSTM consumes 1K epochs, and GAN consumes 48 K epochs for the same task over the same data. This is a known fact [45]. Figure 6 shows the iterative procedure in a more simplistic form; it is less GCN oriented, but it is GCN. There is one major factor that differentiates it from adversary modules. The data provided by the Module 2 “generator” to the Module 1 “discriminator” are usually correct and accurate, and it is more deterministic and not a machine learning architecture. For GAN, the information provided is semi-random. The proposed algorithm system is ever-learning and is shown in Figure 6. First, it trains over a universal dataset [38,45]. Then, it trains over a preliminary 200 m’ local dataset with emulation of frauds on some of the meters, plus 15 verified human detected frauds.

Gradually, as scenarios are tagged as non-frauds, they are tagged using the pooling method as the “anomaly type”, and the training dataset is enlarged. The theory to be investigated is that it is better to split the algorithm operation into three periods, the same as a product lifetime: (1) system infancy stage—twenty verified frauds at the local dataset. It is possible that a “fast learning” algorithm may yield better results experimentally.□

Comment: To provide reference to the previous theorem, there are several reasons why the GAN consumes much more data than the CNN: (i) the GAN requires finding Nash’s equilibrium. There is no systematic algorithm to obtain that in the general case [45]; therefore, this is a difficult task. (ii) Intuitively, adversarial means the generator is tricking the discriminator, which requires more time for the discriminator to converge. (iii) Every time the parameters of one of the models are updated, the nature of the optimization problem that is being solved is changed. This has the effect of creating a dynamic system [46]. (iv) In neural network terms, the technical challenge of training two competing neural networks at the same time is that they can fail to converge [47]. (v) The most challenging model failure is the case in which multiple inputs to the generator generate the same output [47]. In order to avoid that larger dataset is required.

### 2.6. A Robotic Process Automation (RPA) System to ASSIST in Information Loss that Looks Similar to Energy Loss Detection

**Figure 7:**
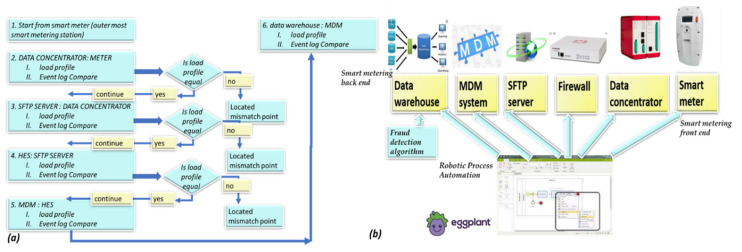
(**a**) RPA sub-algorithm flow diagram. (**b**) Illustration of implementation using the Eggplant [32] software automation platform: comparison of energy load profile and reading meter event log file—enables loss location. Eggplant symbol and GUI is taken from Eggplant website with permission.

Next is a sub-algorithm in the technical/nontechnical loss detection system schematics. Figure 7 describes an RPA algorithm diagram that acts as a secondary TNT loss detector for problems along the data chain, which are initially interpreted as TNT loss. This is due to the work published in [1] showing both experimentally and theoretically that data-chain mismatch signature speaking, based on the energy load profile, looks very similar to the TNT loss signature. Prior to dispatching a technical/non-technical loss detection team to the field, the probability computation must be sufficiently high considering that there are other anomalies. During the infancy stage, the TNT loss algorithm is incapable of differentiating loss types. The simpler RPA algorithm was implemented to enable comparing the load profile and the event log files at various stations along the smart metering data chain from the endpoint, which was the data warehouse. If there is a mismatch between, for example, the data at the meter and data at the concentrator, then that is non-TNT field loss. Otherwise, a comparison is performed in the next station up until the data concentrator for the PLC and head end system (HES) for cellular meters. If everything is satisfactory by that algorithm, then loss suspicion is handled by the sending a team to the field. The closest station to the meter where a mismatch is detected as an anomaly location. RPA accesses any software system as a user with a username and password using a remote desktop protocol (RDP), identifies the graphic user interface (GUI) using optical character recognition (OCR), and performs operations from the GUI menu. Therefore, it is simple to operate and write a code. An RPA is AI; it is a graphic object identification AI using the “recurrent neural network” (RNN) family, specifically named “long short-term memory” (LSTM) [48].

### 2.7. Data Augmentation of Verified Frauds to Fill in the AI Requirement of Scenarios

Due to the imbalance between loss and non-loss, and the lack of verified losses, several known strategies can be used: (1) SMOTE, (2) data augmentation using an autoencoder [49], and (3) finally, data augmentation with five percent white Gaussian noise (WGN). The latter strategy was used. There is a disadvantage to that strategy because if another customer profile behaves differently regarding technical/nontechnical loss detection, it is not clustered. In the following sections, expert knowledge-based feature construction is described. These sections are not replicas of work from paper [1]. They pose a significant feature enhancement serving two objectives: (1) improved TNT loss detection. That was explained as making the signature clusters loss/no-loss farther away through additional expert knowledge-based features. In the mathematical section of the current paper, it is further developed how this affects algorithm performance is explained. (2) For the first time, a system is presented that can identify that there is TNT loss, classify that loss, and is intuitive to comprehend that it is working properly, even before the system is tested in the results section. The GCN nature, which is the TNT loss system core architecture theory, was previously discussed in the work by Ryu et al. [50] and for energy saliency prediction with GCN [51].

### 2.8. The Pooling Mechanism—Second Top Architecture after GCN to Enable Loss Classification

**Figure 8:**
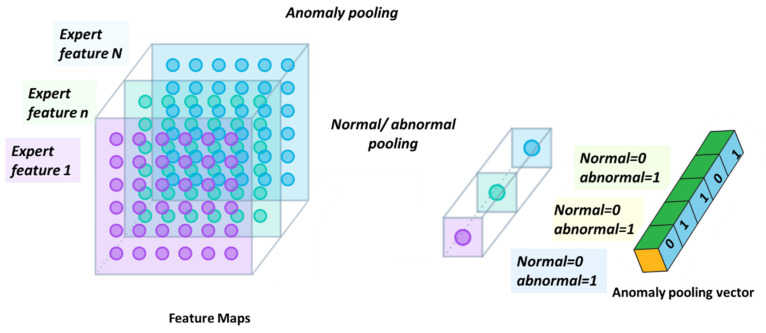
Demonstration of the pooling mechanism.

Described here is one of the mechanisms that provides the proposed “TNT loss” system its power. There is “max pooling” and “average pooling”. The anomaly “pooling principle” presented here and the theory backing it up are new to this paper. There are currently eight expert knowledge-based features, and there is a plan to increase to fourteen in the near future. These are universal features and not consumption based. What makes them universal, as shown in Section 2.8 and what follows, is the “collaboration” of the entire energy load profile capability. There are two implementation options. Figure 8 shows the pooling architecture. Proof that this mechanism works is empirical. The theoretical proof will be the issue of a future paper. Intuitively, if the axes formed by each 3D PCA subspace are fully orthogonal to the rest of the subspaces, this is implied. When the axes are not fully orthogonal to the rest of the subspaces, that still holds. The computational complexity of clustering *N* features collaboratively is by pooling $\;N\times \log\left(N\right)$ and without pooling $\;N^{2}$, similar to the “sparse matrix”. The second option of no pooling needs to learn over an *N*-dimensional space and look for island subspaces where the behavior is normal and anomalous. That requires sufficient data. The feature groups are (i) energy distribution (ii) daily-hourly trends, (iii) seasonal-hourly boxplots, (iv) spectral, and (v) {2D Pearson correlation heatmap} times {active, reactive}—meaning 10 subgroups. Then, the high-order dimensional subspaces are reduced into 3D PCA. Then, they cluster into normal/abnormal. That is normal/abnormal pulling. The pooling technology simply computes a normal/anomalous state to each feature group and generates an ordered vector $\left\{01100100\ldots 1\right\}$, where 1 indicates abnormal. If that is correct, it is simpler and much faster to learn a six-dimensional space than a 1000-dimensional space. It may be roughly proven that for an orthogonal set of features, this is a very good approximation.

### 2.9. Consumption or Universal Expert Knowledge-Based Generated Features

In the current chapter, a large series of features is used to identify that it is loss/no-loss and characterizes a loss-type. Emphasis is prior to using a classifier to obtain two achievements: (a) make the difference between loss and non-loss and technical and nontechnical obvious to human cognition and (b) make the “features space” selection, such that the location of loss and non-loss is nearly binary. We draw a principal component analysis (PCA) 3D feature space and “reduce dimensions” of the original feature space into 3D for visualization and differentiation loss/no-loss enhancement. Binary denotes that the normal and anomalous clusters are far away, such as to enable drawing a linear surface plane that separates two states. That is not performed for aesthetics, it serves several goals that are demonstrated in the results chapter. (i) The verified TNT losses in the initial local dataset are small. When the distance is large, a pseudo-linear classifier will converge quickly based on a few losses tagged as a specific loss type plus a moderate data augmentation module. (ii) It is then possible to draw a conclusion from a low consumption TNT loss into a higher consumption TNT loss of the same type, and vice versa. (iii) It is possible to cluster into specific loss types and subtypes and separate various anomalies that resemble fraud. All the features process the load profile data only, which is a single parameter time series. It may produce up to 256 feature parameters: there is a wise 3D PCA dimension reduction to three. A general rule for a good feature is a collaborative or complete panoramic data view. In [1], it was explained how features containing some new information, which means they have an orthogonal component in feature space, contribute to an enlargement of the distance between normal and anomalous. There is another paradigm shift from that work. There is no insistence on fraud/non-fraud, since the “pooling technology” enables, as later shown, a 1:1 mapping of loss type to a pooling ordered vector. The presented features then differentiate between normal and anomalous, as has been consistent.

#### 2.9.1. Expert Feature 1: Energetic Distribution from the Load Profile

**Figure 9:**
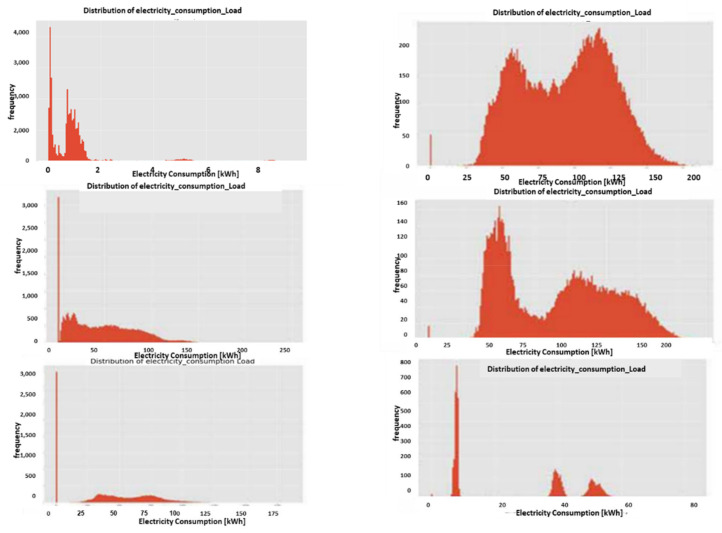
Four verified fraud test cases (two left columns) versus three verified non-fraud test cases. Fraud indicates technical/nontechnical. Technical loss type differentiation is similar.

This feature was previously reported in [1]. The difference now is that it is operable over four energy quadrants: $\left\{active,\;reactive\right\}X\left\{import,\;export\right\}.$ Taking the entire load profile periods and counting the periods at a certain energy level $E_{n}$ and Figure 9:

(5) $$\begin{array}{l} n(E_{m})=\frac{n(E_{n}\leq E<E_{n}+\Delta E)}{\sum_{m}n(E_{m})}\\ n(E)=\lim_{\begin{array}{l} N\to \infty \\ \Delta E\to 0 \end{array}}count\_of(E_{n}\leq E<E_{n}+\Delta E)/\int_{0}^{\infty }n(E)dE\\ n_{cont}(E)=\lim_{\begin{array}{l} N\to \infty \\ \Delta E\to 0,E_{m}\to E \end{array}}n(E_{m})=\frac{\lim_{\begin{array}{l} N\to \infty \\ \Delta E\to 0,E_{m}\to E \end{array}}n(E_{n}\leq E<E_{n}+\Delta E)}{\int_{0}^{\infty }n_{cont}(E)dE}\\ N=\sum_{m}n(E_{m}) \end{array}$$

where:

n is the number of load-profile periods counted with energy that is inside the bin [$E_{n},E_{n+1}]$.
$N=\sum_{m}n\left(E_{m}\right)\;$ is the entire load-profile period count, which is a summation over all bins of period counts. It is not the entire energy, $\sum_{m}n\left(E_{m}\right)E_{m}\Delta E$ is.
$n\left(E\right)$ is a limit continuous function of the series $n\left(E_{m}\right)$ at the point $E_{m}$ when the periods count N becomes infinite and the bins split, ΔE becomes zero.
$n_{cont}\left(E\right)$ is the continuous version of the distribution function according to the energy parameter.

The latter limit ΔE→0 is performed with incrimination of periods count *N* and reduction to zero of the energy step ΔE, because otherwise, the discrete distribution does not reflect the true distribution since there are empty bins that have not yet occurred.

Figure 9 shows the resemblance of the difference between fraud and non-fraud. It is clear that with respect to a person outside the premises, the statistical signature of validated frauds, the peaks are shaved. Regardless of the customer’s disguise, the fraud is evident. That is a very definite feature per fraud/non-fraud classification. It is clear that with respect to validated frauds, the peaks are shaved. That is a very definite feature per fraud/non-fraud classification. Experimentally, other anomalies also resemble this, and loss type identification is performed using pooling technology.

#### 2.9.2. Expert Feature 2: Daily Spectral Energy Distribution

This feature is newly reported in the current paper. The above figure is in the time domain: $n\left(E\left(t\right)\right)$. It resembles a sum of two/three Gaussians. The Fourier transform of a Gaussian is a Gaussian. Now, the time-dependent energy is Fourier transformed based on a daily periodicity. The FFT is performed over ten periods to avoid the “aliasing effect”:

(6) $$E(\omega )=FFT\{E(t)\},\begin{array}{cc} & n(E(\omega ))=n\prime (E)/\int_{0}^{\infty }n\prime (E)dE \end{array}$$

**Figure 10:**
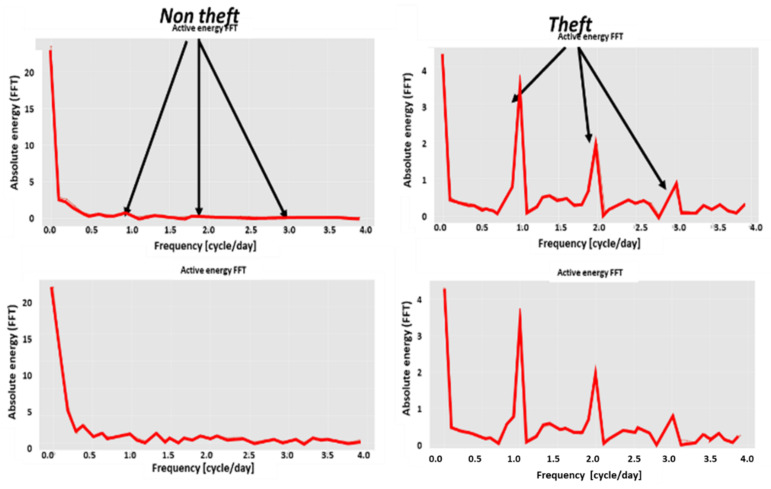
Characteristic non-fraud vs. validated fraud. Fraud—nontechnical and meter-reported failures. Non-fraud—other anomalies. Here, fraud is actual fraud. Technical loss type differentiation is similar.

After FFT, a periodic signal is derived, but only the first period is considered. The results of characteristic fraud vs. non-fraud are shown in Figure 10. Harmonics 1, 2, and 3 are higher for validated anomalies and fraud cases.

#### 2.9.3. Expert Feature 3: Daily-Hourly Trend Graphs:

The next feature is to take a load profile and draw average daily-hourly trend graphs:

(7) $$E_{day(i)}(t\prime =t_{n})=\sum_{period\in t_{n}}E(t\prime =t_{n})/N$$

where

$day\left(i\right)$ $\in \left(week\;days\right)$

$t_{n}$—each day is composed of a fixed number of recorded energetic periods and fixed time periods. For example, in a quarter-hourly load profile: 00:00, 00: 15, …, 23:59, there are 96 quarter-hourly periods. $t_{n}$ is a fixed time occurring each day.

$period\in t_{n}$—summing all periods from each day, one period per day of time $t_{n}.$ For example, $t_{n}=12:15.$

*N* is the total number of days within a recorded load profile, which equals the total number of periods $t=t_{n}.$

$E_{day\left(i\right)}\left(t^{\prime }=t_{n}\right)$—average energy in day of the week $day\left(i\right)$, of the type defined above. Averaging is over all periods of the historic energetic load profile.

**Figure 11:**
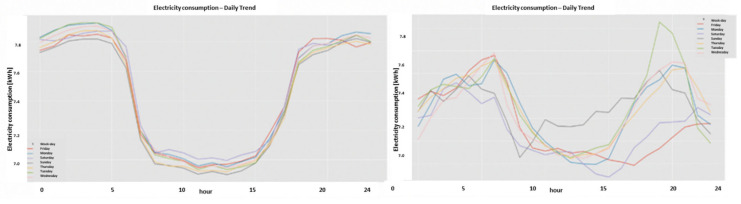
Characteristic daily-hourly trends (0–24) of verified non-fraud (**left**) versus daily-hourly trends of verified fraud (**right**). Here, fraud is actual fraud. Technical loss type differentiation is similar.

Figure 11 shows characteristic verified fraud vs. non-verified fraud and, more generally, normal vs. abnormal. Again, it is visible that non-frauds are separable from frauds. The non-frauds are ordered graphs and are close to each other, and the frauds are unordered. That trend is definite for fraud and non-fraud and other loss/no loss.

#### 2.9.4. Expert Feature 4: Boxplot Hourly Seasonal Trends Acting as a 2D Object Identification System

**Figure 12:**
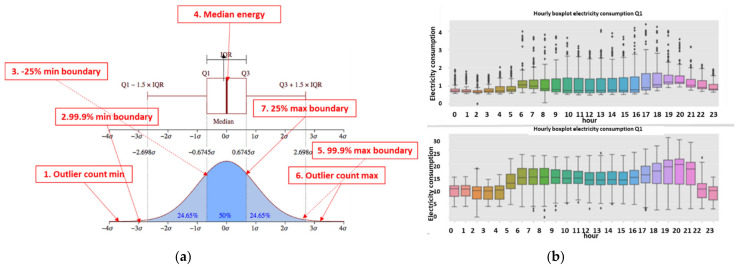
(**a**) Left is the boxplot split into seven features, (**b**) right-upper is verified fraud vs. lower verified non-fraud. Here, fraud is actual fraud. Technical loss type differentiation is similar.

Boxplots [52,53,54] are used for the entire data collaborative description and anomaly detection. The next feature is seasonal-hourly boxplot graphs. Figure 12a describes the correlation between each boxplot and normal distribution through seven parameters that are later used to construct high-order dimensional space. The boxplot graph in Figure 12b right is the most intuitively noticed as a 2D matrix from the rest, and it is used to differentiate between fraud and non-fraud and later between fraud types and anomaly types. It is obvious that there is a differentiation between the validated anomaly and the normality. First, the outliers are counted and statistically increased over the parameters. A boxplot serves as a 2D image and spans over 168 features makes it capable of differentiating between anomaly types—which proved to be true, and justifies expert features. At Figure 12, there are 7 characteristic points selected: (1) outlier count limit – the number of outlier points—exceeding normal distribution center. (2) The 2nd boundary is three times variance, which is about 99.9% of entire results. (3) third point is left boundary of 50% entire distribution. (4) Median energy is the normal distribution mean value. (5) fifth point is three times variance marking the right boundary of 99.9% of entire results. (6) sixth point is outliers count above point 5. (7) is right boundary of 50% of entire population.

### 2.10. Insertion of the Reactive Load Profile to the Learning Space and Comparison to the Active Load Profile

#### 2.10.1. Expert Features: Reactive Load Profile vs. Active Load Profile for Distribution of a High-Order Dimensional and PCA 3D Space

It is worthwhile to investigate how technical/nontechnical loss detection is affected by inserting a reactive load profile compared to active load profile patterns for all the above features and additional features. Not all residential meters have reactive energetic load profiles, but many do.

#### 2.10.2. Expert Feature 5: A “Six-Dimensional” Energy Distribution Space—Reactive vs. Active Energy

A Gaussian mixture model GMM [55,56] fits two normal distributions in the previous Section 2.9.1—the energy distribution feature. An alternative method to GMM is a “kernel density estimation” [55]. For the GMM, the basic equation is:

(8) $$p(\omega )=\sum_{i=1}^{K}\varphi_{i}N\left(\mu_{i},\sigma_{i}\right)$$

where

$\varphi_{i}$ is the peak amplitude parameter, $\mu_{i}$ is the central frequency,
$\sigma_{i}$ is the width, K is the Gaussian count, and  N() is the normal distribution

**Figure 13:**
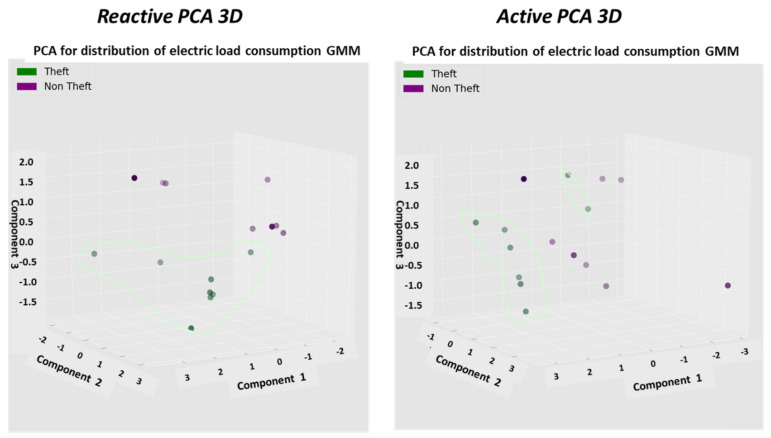
Active (**right**) vs. reactive (**left**) 3D PCA plot of energy distribution as a six-parameter Gaussian mixture model—frauds are scattered green. Non-frauds are dark blue and circled.

A six-dimensional space is constructed and reduced to 3D using the principal component analysis (PCA) linear and orthogonal axes transform [57]. The results are statistical over a two-hundred meters’ local dataset with 2.5% verified fraud, 3.5% phase reversal = technical loss, and phase disconnections, which were considered technical losses. Recall EU-28 [17]. Figure 13 compares active vs. reactive and demonstrates feature effectiveness in each subspace stand-alone. Observations: (a) There is a difference between active and reactive. (b) The non-frauds may be clustered separately from the frauds. (c) This is not so apparent from the 3D plots but is evident from numeric values. The frauds are closer to the $x_{i}-x_{j}$ planes than the non-frauds where i≠j{*x*-*y*,*y*-*z*,*x*-*z*} axis planes. (d) If there is reparability both in active and reactive, then a reactive addition to learning will increase the confidence level. It is similar to two signatures *x* and y. If both appear, then the confidence level is increased. (c) The difference between fraud/non-fraud is binary—that is, an advantage when there are few verified results. First filtration: fraud is nontechnical and meter-reported anomalies and non-fraud are other anomalies. Here, fraud is actual fraud. Technical loss type differentiation is similar. Although various loss types may yield different sections of that space, the ordered vector constructed by the pooling technology makes it simple to distinguish between loss types and reduces training effort = training time and scenario count because it is a binary decision rule. The GMM is alternatively handled by the kernel density estimation method [58] with a Gaussian kernel.

#### 2.10.3. Expert Feature 6: Active vs. Reactive Daily-Hourly Trends

The concept behind daily-hourly trends is the collaborative $L_{2}\;$ distance between curves:

(9) $$\begin{array}{l} h_{i,j(L_{2})}=\sqrt{{\int_{0}^{\infty }\left|p_{i}(E)-p_{j}(E)\right|}^{2}dE/{\int_{0}^{\infty }\left|p_{i}(E)\right|}^{2}dE}\\ h_{\max}=\max\{h_{i,j(L_{2})},i,j\in (regular-week-days)\} \end{array}$$

where:

$h_{i,j\left(L_{2}\right)}$ denotes collaborative distance measurements between two daily curves $p_{i}$, $p_{j}$ i≠j
$p_{i}\left(E\right),p_{j}\left(E\right)$ denotes energetic daily-hourly curves illustrated in Section 2.9.3.
regular−week−days—for Christian-based weeks {Monday-Friday} for Muslim-based weeks {Saturday-Wednesday} and for Jewish-based weeks {Sunday-Thursday}. In general, not including weekends.
$h_{max}$ denotes max-pooling over all combinations of daily trends.

**Figure 14:**
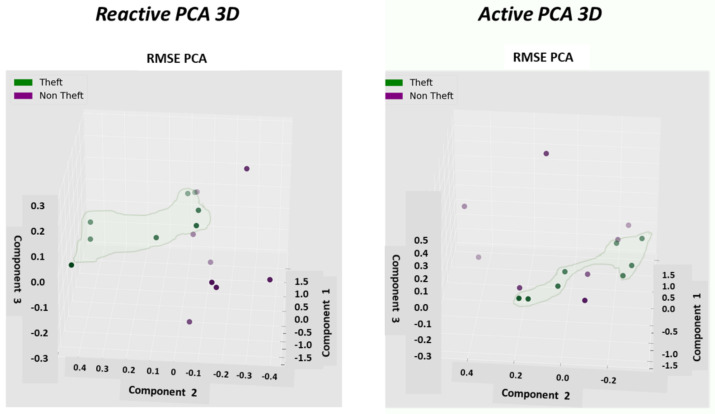
Reactive vs. active 3D PCA of daily-hourly trends RMSE. First filtration: fraud—technical/nontechnical. Non-fraud—other anomalies. Here, fraud is actual fraud. Technical loss type differentiation is similar. The soft green surface is meant to intensify theft cluster.

Figure 14 shows the PCA of daily-hourly trends active vs. reactive. Conclusions: (a) There is a difference between active and reactive loads/customers. (b) The non-frauds may be clustered separately from the frauds. (c) The difference between active and reactive is noticeable, meaning the reactive energy load profile contributes additional information; therefore, it is expected to increase technical/nontechnical loss detection accuracy. (b) Again, frauds are closer than the non-frauds to the $x_{i}-x_{j}$ planes, where i≠j $\left\{x-y,y-z,x-z\right\}.$

#### 2.10.4. Expert Feature 7: Active vs. Reactive Boxplot “Hourly Seasonal” Trends

**Figure 15:**
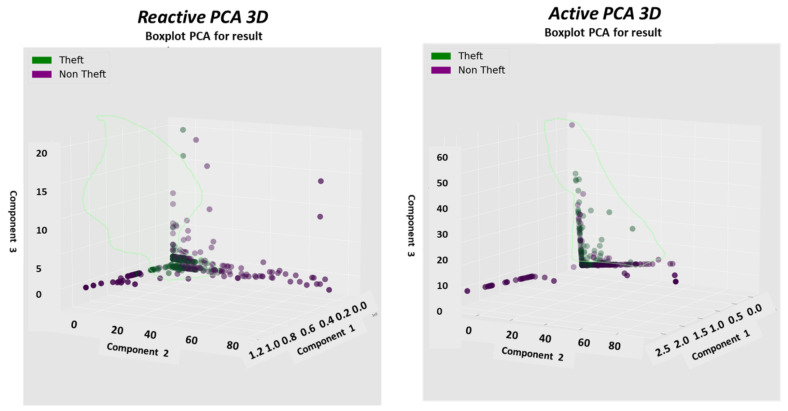
Reactive vs. active 3D PCA of seasonal-hourly boxplot trends—clustered are the non-frauds. First filtration: fraud—technical/nontechnical. Non-fraud—other anomalies, blue cluster. Here, fraud is actual fraud. Differentiation between technical loss types is similar. Soft green transparent reinforces the theft cluster.

Figure 15: From each boxplot, seven points are taken as seven axes. There are twenty-four hours. Figure 15 shows PCA 3D high-order dimensional space smashed from a high-order dimensional space of 168 axes, and there are two subspaces. Eventually, the Pearson correlation heatmap reduces 60% of features, and if operated more strictly, it reduces to the 20 most correlated parameters. The graphs show the following: (1) Strong separability. It is somewhat a little difficult to notice, but the non-fraud is “hovering” above the axes, while three fraud clusters exactly overlap the axes. This is no coincidence and is explained in the theoretical section in the Abbreviations. The theft cloud shows that theft is actually “outside the axes planes while non-theft is overlapping the axes planes.

#### 2.10.5. Expert Feature 8: Active vs. Reactive Pearson Correlation Heatmap

**Figure 16:**
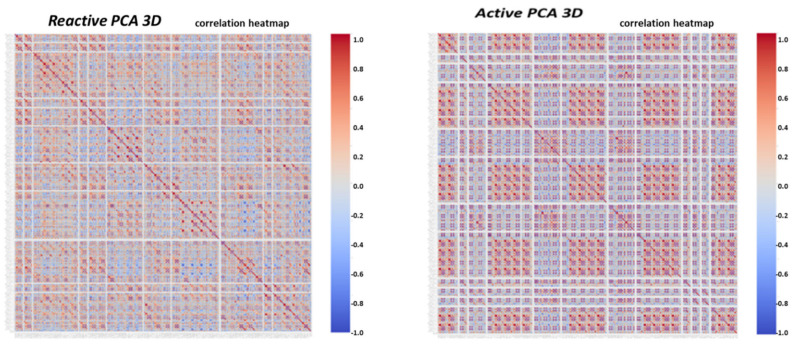
Active vs. reactive—all features vs. fraud, Pearson correlation heatmap. An active map is hotter than a reactive map and is more correlated with fraud. There is no intention of noticing inner variances inside the 2D image. Simple notification of stronger correlation.

Figure 16 shows a correlation heatmap of all parameters to fraud. There are 512 parameters, split between reactive and active. To reduce dimensionality without reducing accuracy, the Pearson correlation heatmap formula is used as a threshold filter to select the parameters correlated with fraud/non-fraud from other parameters:

(10) $$\rho_{x,y}=\mathrm{cov}(x,y)/\sigma_{x}\sigma_{Y}=\sum_{i=1}^{n}\left[(x_{i}-\bar{x}\right)(y_{i}-\bar{y})]/[\sqrt{\sum_{i=1}^{n}{\left(x_{i}-\bar{x}\right)}^{2}}\sqrt{\sum_{i=1}^{n}{\left(y_{i}-\bar{y}\right)}^{2}}]$$

The image is used for two objectives: (a) reduction of 512 parameters only to those correlated. It is selectable and reduces to 40% or to twenty variables. (b) It may be used as a 2D classifier between fraud and non-fraud and loss and no loss. (c) Active is hotter than reactive, which means that most of the contribution to detection is active. This may be a generic result unpublished thus far. The conclusion from Figure 16 is that active energy is much more correlated with fraud.

#### 2.10.6. Expert Feature 9: Reactive vs. Active PCA 3D of All Collaborative Features

**Figure 17:**
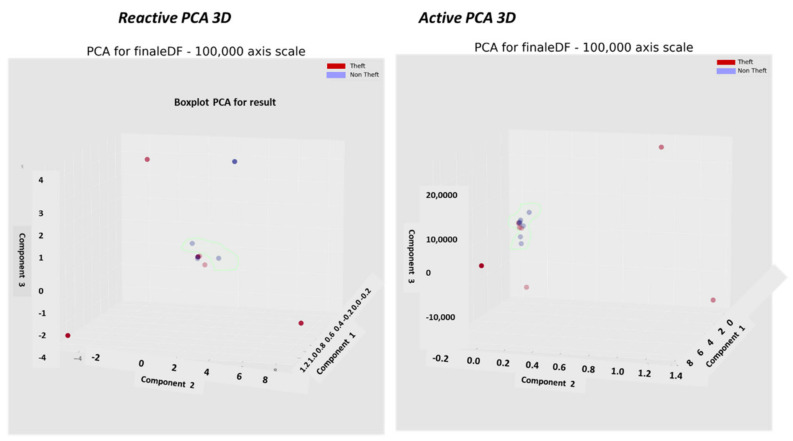
Active vs. reactive final-all features 3D PCA plot. Blue clusters are non-loss, while red clusters are loss.

Figure 17: There are six dimensions from the energy distribution, plus 7 features from daily-hourly trends plus 168 from the boxplot. All are taken to construct a high-order dimensional space. A Pearson correlation heatmap reduces the space by 60% or if desired to twenty features. The original features (count) are reduced in Figure 17 using the PCA Transform. Conclusion: active energy is correlated with fraud, and non-fraud and reactive energy are also correlated with fraud. This means that reactive also contributes to clustering; it is a generic cross-algorithm potential capable of a research conclusion.

### 2.11. A Computational Study on the Effect of Features Space on Training Effort and Accuracy

#### 2.11.1. Forward

The objective of this chapter is twofold. (1) To complement paper [1] Section 2.10 constructed a theory of technical and nontechnical losses. The background on loss detection algorithms is found in [2,3,4,59,60]. That work showed three main issues: (1.1) that the non-fraud or non-loss signature is farther at the “feature space” from the axes than the loss signature, as observed in Figure 10 for example, (1.2) that the energetic distribution of a sum of Gaussians is translated to spectral space to a sum of Gaussians with an inverse width frequency to the time relationship, and (1.3) that using additional features stretches the distance between loss and non-loss clusters of signatures at the feature space. The latter was performed using vector algebra and provided justification for the addition of expert-knowledge features. (2) The objectives of the current paper are (1) to show, using “information theory”, which is an exact quantitative relationship between the feature space and algorithm performance, a dramatic effect in (1.1) training effort and (1.2) the accuracy upper limit. The theory computes only the feature space effect, assuming the classifier stage is trained to approach the theoretical limit derived from the feature space. (2) We suggest a theoretical model for TNT loss “generative cooperative modules” (GCN) by mapping TNT loss variables to a known image processing “generative cooperative networks” model. To explain the physics is complicated and may serve in future enhancements. It must be mentioned that an alternative method to data augmentation used here is imbalanced-learning handling using the SMOTE [61] library, which is not implemented here. It is reminded that features are generated here using this research-generated code library of anomaly detection feature preprocessors. Previous work on supervised learning theft detection was performed by Khan et al. [62].

#### 2.11.2. Computation of the Mix-Up Probability of Two Event Clusters

This chapter explains the mix-up probability computation at the feature space and the accuracy derived from it. A feature space contains clusters of event signatures, with each event per specific scenario of that same loss type. Initially, technical/nontechnical, and gradually, with the accumulation of verified loss types, with the loss types. Pearson’s correlation coefficient (PCC) is defined by the equation:

(11) $$\rho_{x,y}=\mathrm{cov}(x,y)/\sigma_{x}\sigma_{Y}=\sum_{i=1}^{n}\left[(x_{i}-\bar{x}\right)(y_{i}-\bar{y})]/[\sqrt{\sum_{i=1}^{n}{\left(x_{i}-\bar{x}\right)}^{2}}\sqrt{\sum_{i=1}^{n}{\left(y_{i}-\bar{y}\right)}^{2}}]$$

where

$\sigma_{x},\sigma_{y}$ denotes the standard deviation of  x,y
$x_{i}\;$ denotes the forecast object instance
$y_{i}\;$ denotes the actual object instance

This function computes the correlation between these object instances, thereby revealing the correlation in accordance with a specific classification algorithm. The correlation coefficient is in the range [–1, 1] but still may be used as a mix-up probability.

**Theorem** **1.** *A mix-up probability is computable from the feature space.*

**Proof.**  The following transform is performed in [0, 1]. To provide it with probabilistic interpretation, the formula is modified:

(12) $$p(A,B)=\left|\rho_{x,y}\right|$$

Reasoning for selection of the probability function: there are signature instances of type A and of type B. What determines the mix-up probability, or the confusion, is the amount of overlap at the features space, which is the PCC. Three types of clusters may be considered good representatives to comprehend the correlation to the mix-up probability. (1) A device signature cluster with a center and a distribution, which declines toward zero with the distance from the center, never to zero but always declining, (2) a localized distribution and does not exist at a distribution outside a radius $r_{i},$ and (3) a localized homogenous distribution inside a ball of radius $r_{i}$. Which distribution is selected is unimportant in the following discussion. Mathematically speaking, the three distributions are formulated. A homogeneous, isotropic distribution is assumed, which is not always the case but without loss of generality. Homogeneity is obtained with normalization. The desired issue is to comprehend the order of magnitudes and upper bounds.

(13) ρi=ρ0rρi=ρ0r,0,r≥rir≤ri,ρi=ρo0,r≥r,r≤ri

The discussion may be generalized to any world distribution by replacing the equals sign with ≤. Bounding the actual distribution with one of these distributions is sufficient. Continuation of the discussion is performed in a 3D or 2D PCA feature space. The mix-up probability, or PCC, is for the ever declining, never zero distribution is:

(14) $$\rho_{A,B}=\frac{{∰}_{V,N}\rho_{A}(r-r_{a})\rho_{A}(r-r_{b})}{\left(r-r_{a})(r-r_{b}\right)}d^{N}r=\int_{N}\frac{1}{r^{4+N}}d^{N}r=\frac{\rho_{0}}{\left(r_{a}-r_{b}\right)}$$

Epilogue: the distribution has a low effect on the continuing discussion. It is sufficient that the probability is ≤1. □

#### 2.11.3. The Probability of Mix-Up when Classification or Clustering Goes to N Object Types

An algorithm that computers performance parameters for a system capable of identification and classification of *N* fault/loss types is desired. The computation is simple. There may be combinations of *k* loss types active and *N-k* inactive. All combinations are possible while running all possible $O\left(2^{N}\right)$ scenarios. Mix-up probability:

(15) $$P_{mix-up}=\sum_{k=0}^{N}\left(\begin{array}{c} N\\ k \end{array}\right){\left(\rho_{A,B}\right)}^{k}{\left(1-\rho_{A,B}\right)}^{N-k}$$

Now two additional steps are taken. Since the probability is always smaller than or equal to 1, we mark $\rho_{A,B}=1/\left(1+\alpha \right)$. Using the precedence of works [21,22,23,24,25,26,27,28,29,30,31], the information is computed as a figure of training computational complexity:

(16) $$\begin{array}{l} I=H=-\sum_{k}p_{k}\log\left(p_{k}\right)=-\sum_{k=0}^{N}\left(\begin{array}{c} N\\ k \end{array}\right){\left(\frac{1}{1+\alpha }\right)}^{k}{\left(\frac{\alpha }{1+\alpha }\right)}^{N-k}\log\left({\left(1+\beta \right)}^{k}\right)\\ =-\log(1+\beta ){\sum_{k=0}^{N}\left(\begin{array}{c} N\\ k \end{array}\right)k\left(\frac{1}{1+\alpha }\right)}^{k}{\left(1-\frac{1}{1+\alpha }\right)}^{N-k} \end{array}$$

where:

0≤α≤1

$x_{k}$ denotes outcome k of N failure objects.

I denotes information as defined for example in the above references

*H* denotes entropy. The amount of disorder in the cluster.

α equals $\left(1-\rho \right)/\rho$ for ρ≤1 and equals any positive number

(17) $$P_{mix-up}=\sum_{k=0}^{N}\left(\begin{array}{c} N\\ k \end{array}\right){\left(\frac{1}{1+\alpha }\right)}^{k}{\left(1-\frac{1}{1+\alpha }\right)}^{N-k}$$

The reason for taking α is because the true positive mix-up probability moves from 1 at an infinite distance to 0.5 at complete mix-up in the feature space. It does not go to zero. Complete mix-up denotes that there is no way to distinguish between A and B, which indicates 50:50 probabilities for each event. This is not the end of the computation. The two series in Equations (16) and (17)- are further computed. The binomial expansion of ${\left(1+q\right)}^{N}\;$ is used, and the series is replaced with the compact expression. The information formula is identified as a derivative according to $x=\left(1/\left(1+\alpha \right)\right)$. The two series in Equations (16) and (17) are further computed:

(18) Pmix−up=(1−11+α)NI=−log(1+β)N(1−11+α)N−1

The information *I* represents the algorithm training effort: scenario count and training time. The result that appears absolutely non-intuitive is intuitive. When there is mix-up, a synonym for clustering disorder, there is more information to learn and more training effort, only to attempt to remain at a predetermined accuracy. This is intuitively comprehended according to the two corner cases. The above Formula (16), as tedious in detail as it is revealing, is a simpler structure of multiples of separable variables:

(19) $$\begin{array}{l} I=\sum_{k=0}^{N}f_{1}(\alpha ,k)f_{2}(N-k,\alpha )\\ P_{mix-up}=\sum_{k=0}^{N}g_{1}(\alpha ,k)g_{2}(N-k,\alpha ) \end{array}$$

To know that the obtained Formulas (15) and (16) are correct and in the right direction, the two corner cases of “grid analytics” algorithms are analyzed where, in these corner cases, alternative computation reasoning may be applied.

In the case of the infinite distance between signatures in the feature space, there is no mix-up: $\rho_{A,B}=0,\;\alpha =0$. The variable α is set to $2^{-N}$ to find the limit value:

(20) α=0:I=2−NN2N−1=O(N/2)α=0:P=0α=1:I=log(2)⋅N⋅(32)N−1α=0:Pmix−up=12

In the case of complete overlap, the mix-up probability is 1/2 as there is no way to know whether the event is A or B. It is important to note that probability is declining to 0.5. If it remains at a single pair A,B, that generates a correlation and information heatmap.

**Theorem** **2.** *Complete mix-up: proving in an alternative way that the required number of scenarios to train the worst case requires* $\;O\left(2^{N}\right)$ *scenarios.*

**Proof.**  For N signatures, there are $2^{N}$ possible combinations of $\left\{{\mathrm{event}}_{1},{\mathrm{event}}_{2},\ldots .,event_{N}\right\}$, where an event is a Boolean variable of occurring/not occurring. When there is no discount, the signature is the event-type cluster collaborative signature, and the only method is to let the machine study that signature. □

**Theorem** **3.** *No mix-up at all: proving in an alternative way that the required number of scenarios to train the algorithm when the events in the feature space are infinitely distant is*$\;O\left(N\right)$.

**Proof.**  When the distance is infinite in the feature space so that the accuracy is one, then a linear surface in the feature space is sufficient. That is, $O\left(1\right)$. When there are *N* events, then that is *O(N).* □

Epilogue theorems 1 + 2: (1) The universal equation system represented by Equation (18) converges in two corner cases to the results obtained for these cases by another reasoning. That is very unlikely to be a coincidence. The results explain the effect of adding expert knowledge features beyond the vector algebra explanation of work [1]. They also show the dramatic effect of feature generation on algorithm performance: accuracy and training effort. An exponential effect demonstrates an alternative approach to the implementation of stronger and more powerful deep learning algorithms. Instead of going through the classification problem using brute force, the wall is softened by making the distance between clusters larger and then penetrating the wall. (2) Another second powerful technology named pooling also dramatically reduces the training effort by simultaneously training over small dimensional spaces. (3) Third is the generative cooperative modules (GCN) discussed below, which by using three methods, reduces the training time: cooperative vs. adversarial, simultaneous training of the generator and discriminator over the same dataset, and Module 1: generative being deterministic and not speculative. (4) The fourth method uses data augmentation. It does not decrease the scenario count, but it decreases the original scenario count.

### 2.12. Generative Cooperative Modules Theory

It is interesting to provide a theory of generative cooperative modules. The theory is presented by identifying a suitable theory from another discipline, computer vision, and mapping 1:1 TNT loss variables to that theory. There are many works on generative adversarial networks. There is much less material on “generative cooperative networks” (GANs) and on “multi-agent cooperative networks”. It is not intended to turn this into an article within an article but rather to introduce the mathematics of cooperativeness. GAN is a zero-sum game between two agents: one representing, for example, the electricity utility company and the other attempting to mislead, which represents the customer. Cooperative is not governed by zero-sum and reaches Nash’s equilibrium. At CoopNets, other policies dictate progress toward equilibrium. The best-described theoretical models found are “generative cooperative networks” (CoopNets) [13,14] and “multi-agent actor-critic networks” [50], each representing another point of view on the same mechanism. The motivation for this section includes (i) the existing works describing networks; here, they are simply AI modules of classical machine learning due to faster convergence. The convergence algorithms have a similar effect. (ii) Previous works have been developed for computer vision. The presented work is developed for TNT loss detection with its specific interpretation and semantics. (iii) Understanding the dynamics behind the presented architecture assists in comprehending the mechanism. If not zero-sum, then “consensus” is the mechanism reaching equilibrium, and the Langevin equation may provide the physics. Future work will benefit from comprehension. The most suitable work that was performed to this paper’s work, is work by Jianwen Xie from UCLA on “cooperative training of discriminator and generator networks” [13] and by Korean Heechang Ryu on “multi-agent actor-critic generative cooperative policy networks” [50]. The current presented work does not train for image processing but on “loss type detection and classification”. The paper‘s architecture is not deep neural networks and is not dependent on the theory presented in work [13]. However, the dynamics of such a system are well suited and described in [13,14], including the Langevin equation. CoopNet theory that is adapted to TNT loss is presented here.

### 2.13. Generative Cooperative Module Theory Applied to Technical Nontechnical Loss Detection—A Classification Problem with a Generator and Discriminator

There are several guiding rules for convergence to equilibrium: (1) minimum energy (effort) maximum likelihood, (2) latent variable and maximum likelihood. The cooperative model is the Langevin dynamics equation. There is a two-step construction of the model: (1) generative modeling of AI modules—presentation of the architecture, and (2) a theory of generative highly collaborative entire data AI modules [14]. The theory is adapted from convolutional neural networks. The difference from CoopNets is that there is no mathematical dictation of the backpropagation algorithm, but this is the nature of the occurring process. Let $p(\vec{Y}|\vec{\theta })$ be the distribution model of the loss types:

(21) $$p(\vec{Y}|\theta )=\frac{1}{Z(\vec{\theta })}e^{f(\vec{Y}|\vec{\theta })}p_{o}(\vec{Y}),Z(\vec{\theta })=\int p(\vec{Y}|\vec{\theta })d\vec{Y}$$

where $\;\vec{Y}-$ is the vector of the loss type, such as “phase disconnect”, “phase reverse polarity”, “magnetic tampering”, “information loss localized at the interface between MDM and the data warehouse”, and many more objects of the bi-vector type {loss type, loss location, meter type: manufacturer, type: {single/3-phase direct/CT/CT-VT connected}. The meter type affects the most likely decision per specific loss event alert. Type *A* may be a very reliable loss alert, while type *B* may be a false loss alert due to a failing meter’s sensor.

$\vec{\theta }$ collects the known parameters to be learned from the data: electric load profile expert-knowledge features, such as {energy distribution, and daily-hourly trends}. We take this as the final processed data “expert feature LEDs positive/negative” defined later in Section 3.3.

The distribution model was developed by Dai and Wu [14] for generative networks.

$Z\left(\vec{\theta }\right)$ denotes the normalization constant to reflect the distribution function.
$p_{o}\left(\vec{Y}\right)$ is the reference distribution common to all loss types.
$f(\vec{y}|\vec{\theta })$ denotes the scoring function for a class Y of {loss type, loss location} conditioned with unknown parameters to be learned

A question is raised as to why to select the distribution defined in Equation (21). An exponential distribution has thermodynamic reasoning. Thermodynamic reasoning relates “entropy to statistical distribution” and “entropy to energy”. Thus, the classical thermodynamic definition transforms into the modern information theory definition, using the same physical rules. This is not surprising since classical thermodynamics is based on particles and information thermodynamics on objects. Thermodynamically, they share the same rules. Let $p_{o}\left(\vec{Y}\right)$ be the reference distribution of any loss type, and the component that is not “loss type” dependent be a normal distribution around the zero mean:

(22) $$p_{o}(Y)=\frac{1}{\sqrt{{\left(2\pi a^{2}\right)}^{D}}}e^{\left[-\frac{{\left\|\vec{Y}\right\|}^{2}}{2a^{2}}\right]}$$

where:

*D* denotes the dimensionality of {loss type, loss location}.
α denotes the distribution variance.
$\vec{Y}$ is the vector of the loss type, such as “phase disconnect”.

It makes sense that each loss has, according to reasonable assumptions, a normal distribution around a nonzero value $N\left(\mu ,a\right)$ that may be normalized to zero.

**Figure 18:**
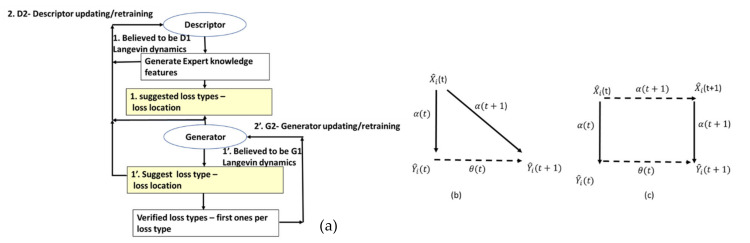
(**a**) Flowchart of cooperative training performed by the discriminator and generator over the same self-generated dataset. (**b**) The teaching procedure of TNT loss follows the MCMC teaching procedure probabilistically, although no Markov chain and no Monte Carlo are intentionally constructed. (**c**) A more elaborate Markov chain describes the same cooperative training.

It is now shown how the algorithms for the generator and discriminator are mapped 1:1 to the model described in [13,14] for CoopNets. The cooperative training flowchart is shown in Figure 18. Simultaneous training of both modules saves time compared to non-simultaneous training and to GAN adversary networks. Explaining Figure 18a: The generator generates a suggestion based on electricity events arriving initially from the meter and on expert knowledge-based assertion rules, operated on measurements from the meter. The same suggestion, based on the same timestamp, is analyzed by the discriminator. This generates expert knowledge-based features over the electricity load profile and four parameters from customer textual data. To save time, both modules/networks learn from the same dataset. After a team is sent to the field to validate or reject a new loss (type, loss location) or non (type, location), the object is added as the scenario procedure repeats.

There are two time frames. In the short range, only two separated AI modules exist: generator, discriminator and long range, with feedback loops as they become cooperative. Figure 18b is more complicated to comprehend. Initially, com $\vec{Y}$ is a vector of loss type and loss location. θ  collects the known parameters to be learned from the data: electric load profile expert-knowledge features, such as {energy distribution, daily-hourly trends, may be short written as the alert LEDs defined in Section 3.3 at Testing sections. α is the statistical dynamics of the change in samples, which we mathematically demonstrate to be the Langevin equation, which is understandable. Variable X is the latent vector latent to the dataset, factors, and variables that are not directly observed but are rather inferred (through a mathematical model). Some complex loss relations are less known, and the remainder may be represented as a normal distribution signal. For example, energy consumption is affected by a vector {weather, historic load profile, tariff program} and more implicitly by {weather, psychology social profile, tariff}. As a reminder, the current paper does not span the entire presented work in [13]. It is only satisfied by showing how the model presented in that paper for visual recognition fits as a generic model to the TNT loss detection architecture. Figure 18b demonstrates how the unknown implicit vector affects the measurements through the Langevin statistical distribution dynamic equation [51]. The Langevin equation is related to generative cooperative networks, an equation of the ensemble of “particles” here {loss type, loss location} scenarios. The dashed arrows represent an explicit deterministic shift from $Y\left(t\right)$ to step $Y\left(t+1\right)$, with explicit known variables θ. Figure 18c shows a more robust architecture where latent factors are also time shifting. There is no end to approximations because latent variables *X(t)* are known to be dependent on several previous steps t, t−1, t−2, and there may be second-order dual-step dependencies. The main theme is comprehended, which is the suitability of the “generative cooperative networks” theory from computer vision to TNT loss. In addition, that theory is not simple.

## 3. Results

The expert knowledge-based feature generation preprocessor was cascaded with several machine learning classifiers and compared with other works.

### 3.1. A Comparative Experimental Study of the Proposed Expert Knowledge Preprocessor with Various Clustering Algorithms Compared to Other Works

Table 1 lists running the preprocessor in cascade to various classifiers, a support vector machine, ridge, random forest, decision tree, and logistic regression, compared to previous works.

### 3.2. Detection of Ten Technical/Nontechnical Losses and Faults Using TNT

The implemented generative cooperative network (GCM) system was run on the smart metering system, and the following sample events were detected using pooling technology. This chapter of the research demonstrates how the generative cooperative modules can accurately identify the fault with relatively less training data compared to other methods. It implements the technical loss section of the “TNT loss” system. Actually, this architecture also enables the classification of nontechnical loss types. Nontechnical loss types non-overlap with technical loss types, such as magnetic tampering, and overlap such as reversed-phase and disconnect phase. Table 2 lists eleven verified loss types, all generated by the presented “TNT loss” system2014a success beyond expectation.

It is accepted that high-quality expensive meters rarely have such field failures as a faulty sensor, but at smart meters’ tenders, the lower bidder may win, and there are inexpensive meters that are of good quality after a cycle of defect handling. The next sections show the AI signature of each event. The following sections have several objectives: (i) the signatures are shown to exemplify that prior to dispatching a team of testing people to the field, then at the current level of research, the AI signature can distinguish whether this is a technical or nontechnical loss. Alternatively, it is a false event, generated due to a meter’s false sensor, for example, obviously at quantities smaller than 0.05% of the meters’ population. The pooling technology on which this work bases the grounds of 1:1 mapping to loss type; (ii) at the other layer, it is shown for four events how they are computer-vision differentiable by the AI expert knowledge features, not just fraud/non-fraud or technical/nontechnical losses; (iii) finally, a classification map of several events is shown. Several issues are observed in the following section: (1) the success of the GCN architecture in the identification and classification and allocation of so many loss types, with much room for new types; (2) the number of examples it would take for an AI to solely study the load profile signature and to identify and classify each loss type; (3) the inability to identify nontechnical and technical losses if the algorithm cannot identify technical losses due to their similarity; (4) the collaboration of two modules’ ability to remotely learn about loss type. Pooling semantics: Here, an alert LED is assigned to each expert knowledge feature. The idea is demonstrated here on four expert knowledge features. When an abnormal LED is on, it is marked as □ to symbolize a turned-on alert LED, signifying anomaly according to the matching feature. When the normal/abnormal LED is off, it is marked as ▀. A set of experiments demonstrates a different AI LED signature for every event. For four features, there are 16 possible signatures. For 14 features, there are 16,384 features. The four features are in the following order: {energy distribution, seasonal-hourly boxplot, and daily-hourly trends, scatter hourly graph}. It is demonstrated here that providing that there are sufficient features, it is extremely deterministic to obtain a unique alert LED signature per problem; that is, even before the AI classification and before plenty of other information. That LED alert technique accelerates the training.

### 3.3. Test Case 1: Power Quality Events

**Figure 19:**
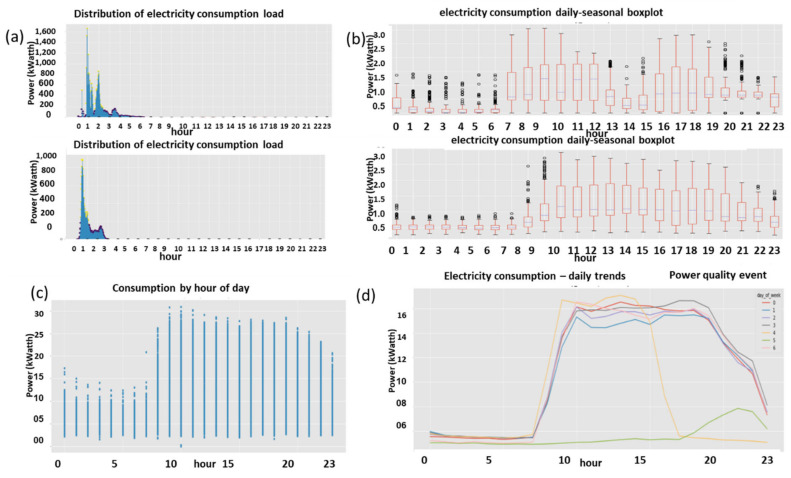
Sample feature graphs of Module 1 when there is an abnormal multitude of power quality events. (**a**) Energetic distribution, (**b**) seasonal-hourly boxplot, (**c**) hourly scatter, (**d**) daily-hourly trends.

This is a reference case of no technical or nontechnical losses. Alerts are of a multitude of power quality events at a specific premise. The events are not supposed to violate the metering correctness. Figure 19 shows that the energy distribution is not smooth. Observation shows the seasonal boxplots contain outliers. The daily-hourly trends are ordered, yet extremely interesting. There are three weekend days.

The scatter plot is relatively clean of outliers. The game plays as follows: Module 2, the suggestion module, suggests an anomaly due to excessive “power quality events” and “TNT loss”. However, it does not know whether there are excessive power quality events at the premises or whether the sensor is broken and whether it affects metering. The other entity “Module 1” is the AI that analyzes the energetic load profile. All the features separately “initially alert” about “normal behavior”, namely: (a) energetic distribution is “thick graphs” rough sum of Gaussians, (b) seasonal boxplots are mostly boxes, with minimal outliers count, (c) scatter is the same, mostly continuous lines, with minimum outliers, and (d) daily-hourly trends are minimal. They are close to each other and ordered. All eight feature flags yield “normal”. Therefore, the TNT loss system conclusion is a normal non-loss scenario. Possibly excessive power quality events originate at the premises. The designed features are numerically quantized, so it is not necessary to analyze the graphs. Using the features as alert LEDs ▀ ▀ ▀ ▀ = {0000}, all alerts are off. As a result of normality: the energetic distributions at Figure 19a, are roughly two Gaussians as described at Section 2.9.1. The daily-seasonal boxplots are with relatively moderate outliers (dots) count as observed at Figure 19b. The average daily-hourly consumption is nearly without outliers as observed at Figure 19c. The daily-hourly trends are completely smooth and similar during week-days, except for week-end. Power-quality events are not theft events and therefore all indicators are normal. 

### 3.4. Test Case 2: Magnetic Tampering

**Figure 20:**
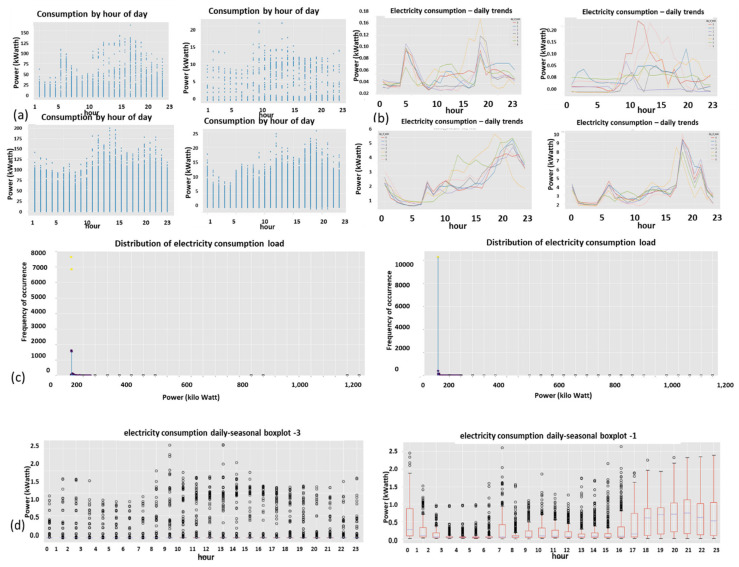
Sample features generated by the module. (**a**) Scatter plots, (**b**) daily-hourly trends of the same occasions. (**c**) energy distribution, (**d**) seasonal-hourly boxplots.

Figure 20 describes the features generated by Module 1: scatter graphs show an excess of outliers for the upper graphs of (a) and different amounts of abnormality. The upper left is a weaker anomaly than the upper right. Group (a) at the bottom left and right appears to be normal and (b) the same conclusions are reached for the daily-hourly group. The upper graphs are disordered. A weaker disorder is seen on the upper left than on the upper right, and (c) the energy distribution graphs reveal the same indication, the upper two are technical or more likely a nontechnical loss. The bottom two are possibly faulty meter sensors, as verified in the field. (d) Seasonal-hourly boxplots. The same conclusions were drawn. Eight virtual sensors generated from the energy load profile indicate the same phenomena. Using LEDs as alert lamps: □ □ □ ▀ = {1110}. Three alert LEDs are on, while fourth is off. Figure 20: (b) daily-hourly trends, (c) energy distribution f(E), (d) (left) electricity consumption hourly scatter plot, (right) electricity consumption daily-seasonal boxplot.

### 3.5. Test Case 3: A Single Phase Disconnects

**Figure 21:**
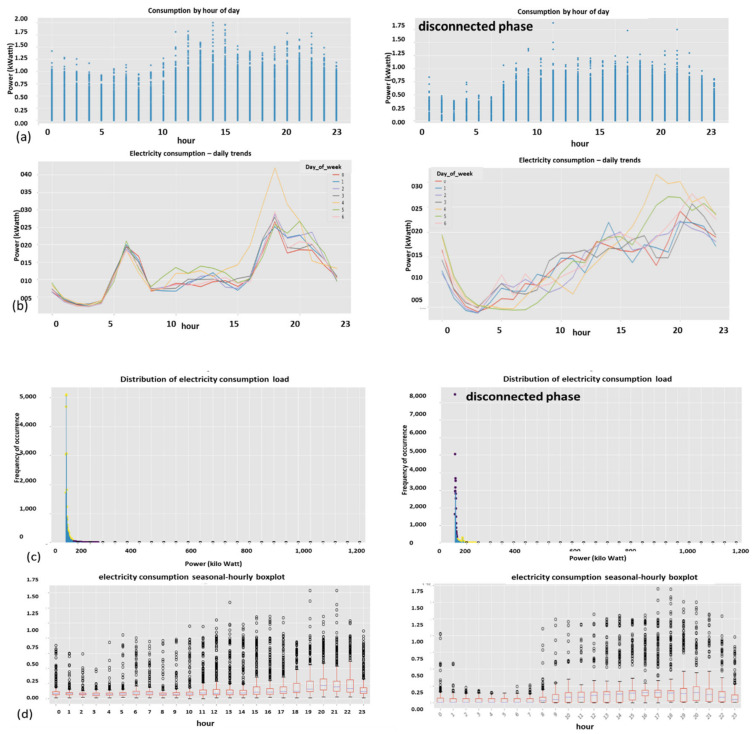
Phase disconnect: (**a**) scatter, (**b**) daily-hourly trends, (**c**) energy distribution, and (**d**) seasonal boxplot.

The phase disconnect is detected by three alerts by the two networks: (i) a phase disconnect event alerted by Module 2, (ii) the assertion rule: voltage≠0, current=0 is triggered, and (iii) the anomalous features of Module 1 AI. Observing Figure 21, the AI Module 1 sample features demonstrate that there is an abnormality: (a) seven incidents have relatively ordered daily-hourly trends. This feature shows a difference from magnetic tampering, which enables the AI to eventually differentiate between loss types at Module 1, the AI level. Reasoning: a single phase discrepancy is ~30% of the total consumption, while magnetic tampering is closer to ~100%, (b) the hourly scatter graphs also do not show anomalies, and there are not too many outliers. Again, ~30% is an issue, (c) seven incidents out of seven alerts have shaved energetic distributions, (d) seven incidents of seasonal-hourly boxplots have plenty of outliers Using features as alert LEDs: □ □ ▀ ▀ = {1100}.

### 3.6. Test Case 4: Smart Metering Data Chain Failure—Load Profile with Gaps

**Figure 22:**
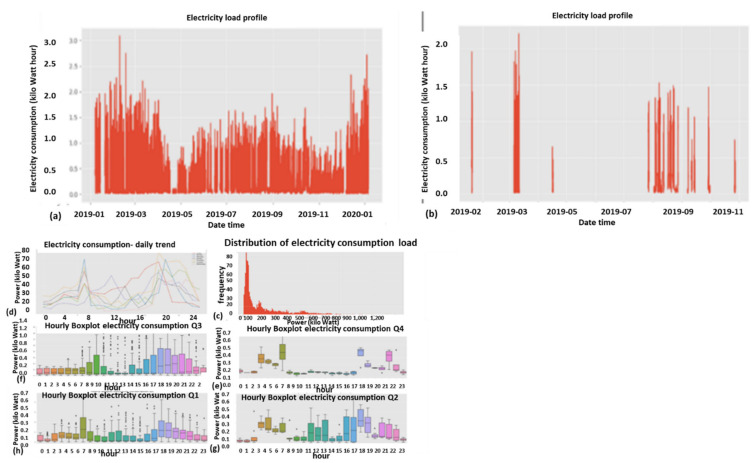
(**a**,**b**) Upper load profile does not propagate fully to the data warehouse, (**c**) shaved energy distribution, (**d**) daily-hourly trends are messy, (**e**–**h**) seasonal-hourly trends with plenty of outliers.

Figure 22 shows the anomaly detection of a load profile with gaps as analyzed from expert knowledge features. Here, the entire spectrum of features is anomalous. There is no suggestion from Module 2: the field with regard to abnormality, but there is no doubt that the data are faulty. The RPA “failure localizing system” presented in Figure 7 determines the failure location. Had the problem been routed to the premises, it would have been a technical loss at the meter location. Root cause: the problem was generated due to imperfect load profile transfers from the meter data management system to the data warehouse (DWH). That problem is information loss. The solution to the problem was implemented by using a handshaking monitoring system named the TALEND “data integrity and governance” system. That system performs handshaking of the data transfer. The problem was allocated using the robotic process automation system comparing meter data between the data warehouse and MDM once information gaps are detected at the DWH. Using the expert features as alert LEDs: □□□= {111}. Observing the graphs: there is no doubt of abnormality, and the RPA allocates the loss or mismatch location by load profile comparison. At Figure 22 top two figures marked as (a), (b) are energy load profile: left is at metering data management system (MDM) the other truncated is at the data warehouse. Figure (a) bellow is daily-hourly trends, Figure (b) energy distribution. Figures (e)–(h) are seasonal hourly trends Q1–Q4. Information loss is observed herein at top at the energy load profile. The anomalies are turned on as if it is energy loss. 

### 3.7. Test Case 5: A Meter Internal Multiplication Factor Attenuation

**Figure 23:**
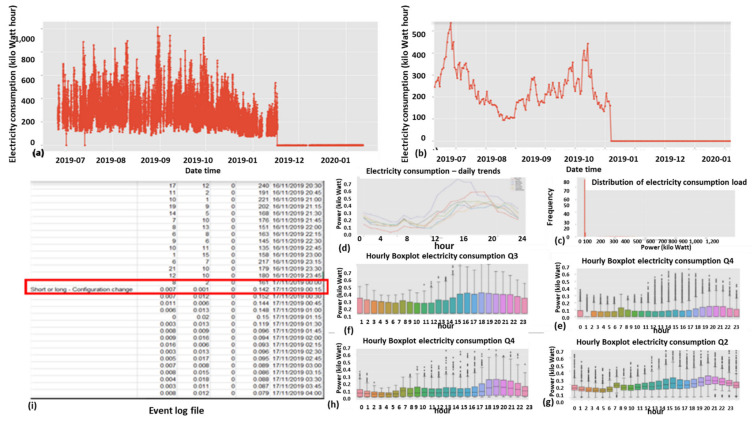
(**a**) Load profile at MDM, (**b**) load profile at the data warehouse. The load profiles are equal, (**c**) energy distribution appears shaved, (**d**) daily-hourly trends look normal, (**e**–**h**) seasonal-hourly boxplots Q1–Q4 appears continuous but with plenty of outliers. (**i**) event log with timestamp marking of the start of the anomaly.

Figure 23 shows expert knowledge-based features of a meter with firmware origin multiplication factor issue occurring only for one out of 10,000 m. Marking features as alert LEDS, they are ▀□▀□□□= {0101000}. This regularity may be used to identify the phenomena, and a preliminary major vote 3:2 when the problem is encountered for the first time using expert features as alert LEDs: ▀ ▀□□= {0011}. This is a more definite loss-type signature than anomaly detection. That problem is information loss.

### 3.8. Demonstration of the Robustness of Expert Features to TNT Loss Determination

**Figure 24:**
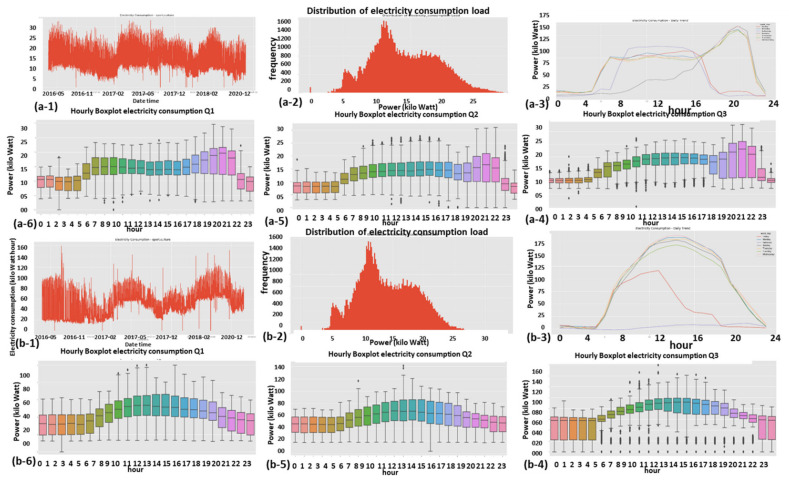
Two characteristics of non-fraud examples. Left to right: (**a-1**) load profile, (**a-2**) energy distribution, (**a-3**) daily-hourly trends, (**a-4**) Q3 seasonal-hourly boxplot, (**a-5**) Q2 seasonal-hourly boxplot, (**a-6**) Q1 seasonal-hourly boxplot. All graphs indicate normality. (**b-1**–**b-6**) same graphs for another meter.

**Figure 25:**
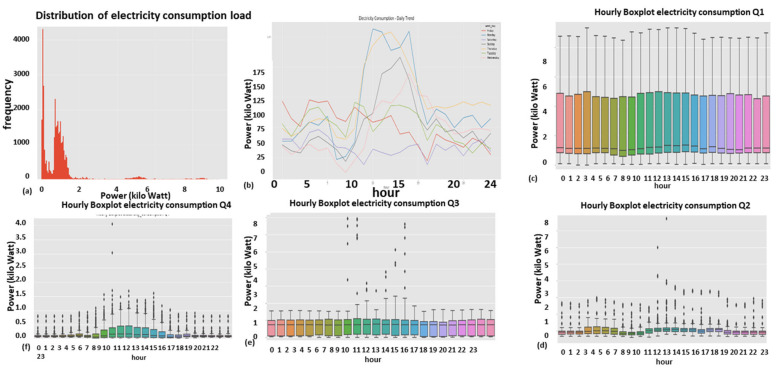
A characteristic of fraud examples. Left to right: (**a**) Energy distribution; (**b**) daily-hourly trends; (**c**) Q1 seasonal-hourly boxplot; (**d**) Q2 seasonal-hourly boxplot; (**e**) Q3 seasonal-hourly boxplot. (**f**) Q4 seasonal-hourly boxplot. All figures indicate abnormality.

Figure 24 demonstrates non-fraud over all characteristic features. Figure 25 demonstrates characteristic fraud as observed by expert-knowledge features. Using expert features as alert LEDs: ▀ ▀ □ ▀ = {0010}. There are characteristics differentiating from non-fraud, the daily-hourly trends are disordered, the energy distribution heights are shorter than the widths, and the boxplot consumptions have a sub-consumption outlier b above normal consumption. The load profile is smooth. The conclusion is that if all signatures are characteristic of their class, then all frauds and all non-frauds may be classified separately. Using the expert features as alert LEDs, □□□□ = {1111}.

### 3.9. A comparative Study of Smart Metering Failures and Technical/Nontechnical Loss Events—Can They Be Differentiated

**Figure 26:**
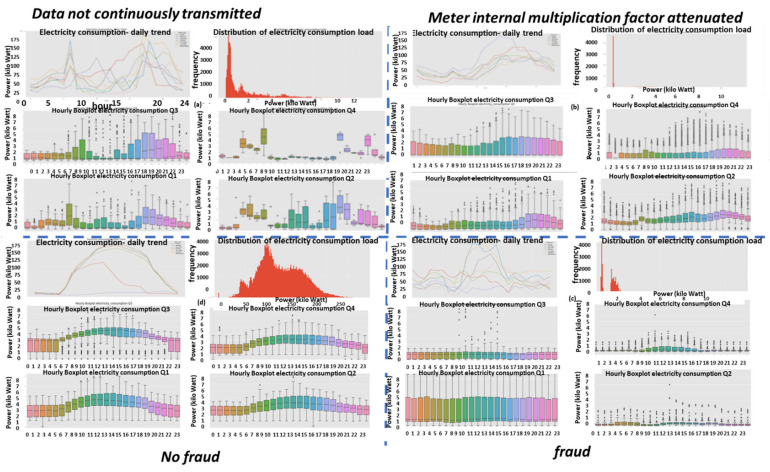
Characteristic signatures. Top left clockwise to bottom left signatures of (**a**) “data not continuously transmitted from MDM to data warehouse” failure, (**b**) meter internal multiplication factor attenuated due to firmware bugs, (**c**) verified fraud, (**d**) verified non-fraud.

Consider that RPA may detect cases (1) and (2) and separate between them regardless of the proposed TNT loss AI system. However, the “fraud system” is also distinguished, as is shown here, due to a multitude of features generating a 2D pattern. Based on the pooling vector, the loss types may be mapped 1:1 to the vector. Observation of characteristic differences over the same features among different loss types is presented next. Observing Figure 26.

(i) Recognition-like AI of the graphs. Two failures occurring in the field out of many in a stable smart metering system are compared to fraud and non-fraud to determine whether separability between anomaly types is possible. Figure 26 shows characteristic signature graphs. It may be understood that non-fraud and no anomaly are separable. Data mismatch failure (i. 1). The daily-hourly trends (1-a) look much messier than the remaining cases. (i. 2) The seasonal-hourly trends (1-c to 1-f)—if the boxplots are considered without outliers, then the “wavy” nature is broken into a discontinuous shape in Q1, Q2, and Q4. This is unique to anomaly (1). (i. 3) The outliers indicate anomalies for cases (1)–(3).
(ii) Observing case (2)—meter internal attenuated due to a firmware bug during the daily time sync by the server network time protocol (SNTP). (ii. 1) The daily-hourly patterns (2-a) are not ordered, but they are the tidiest among all anomalies. Because the consumption pattern is not violated, (ii. 2) the outliers (2-c to 2-f indicate an anomaly, (ii. 3) the energy consumption distribution (2-b) is smaller than the fraud, (ii. 4.) the variance between quadrants Q1–Q4 (2-c to2-f) is much sharper than in the fraud case. There are sub-consumption outliers below the boxplots similar to regular consumption (2-c to 2-f), unique to case (2).
(iii) Observing the fraud in case (3) is easily separable from cases (1) and (2).
(iv) Finally, in case (3), the daily-hourly trends (3-a) are messier than (4-a, 2-a). The outliers (3-c to 3-f) indicate anomalies and are much more intense than in case (2). The energy consumption (3-b) is larger in the case of fraud than in case (2). The quadrants Q2, Q4, and Q3 boxplots (3-c to 3-f) look similar. The boxplot wave is smooth and not wavy. There is no need to implement all these rules using the software. The classifier and feature space should use them.

The conclusion from Figure 26 is that a classifier AI with sufficient examples in the “steady-state” stage is capable of distinguishing between all these cases. They reside in different clusters. The LED alert technology, named “pooling” for AI scientists, shows how deterministic it is to the loss type. It accelerates the learning since experience shows that it does not take too many validated scenarios to come to the conclusion as to what is the correct decoding.

### 3.10. Techno-Economic Impact Analysis and Its Implied Future Research

The statistical EU-28 benchmarking report [17] surveyed 28 countries. The average European energy loss is 7.5% in the distribution grid and 7.5% in the transmission grid. The local example is an OECD country. The proposed system is operable only in the distribution low voltage grid. There are 250,000 residential smart meters and 60,000 industrial smart meters deployed. The national plan is to deploy 3.2 million smart meters up to 2029. That covers 100% of customers. Based on current meters, the distribution grid detected energy loss is 6%. Extrapolated on full deployment and on energy market of 7.065 B Euro expectancy is of energy loss detection of 0.424 B Euro. The transmission grid is not covered by the current system, although it is covered with smart meters. The 60,000 industrial customers consume 66% of the local national energy. The present energy loss detection is 0.282 B Euro. The Italian smart metering 1st generation deployment, which is currently deploying the 2nd generation, relies heavily on TNT loss detection [63,64,65]. To be adapted to the transmission grid, there are two future planned algorithmic changes: (1) Additionally, planned transmission grid consumption expert knowledge-based features based on talks with field engineers. These features focus more on current, voltage and impedance time series. However, these will not suffice in our group’s opinion, and it is planned to rely on the P1 DSMR port of the national local smart meters, emitting a 28 electric parameter load profile every 10 s [66] implemented according to the European Union 2nd channel regulated for 2nd generation smart meters [17]. (2) The P1 DSMR port should also enable the implementation of power flows at many more endpoints than the local company Siemens advanced distributed management system (ADMS) project for power flows. The power-flow algorithm may be used for TNT 5 loss detection. This will enable the improvement of the TNT loss in the transmission grid but also in the microgrid and distribution grid. That future enhancement is expected to squeeze the forecasted 15% of TNT loss.

## 4. Discussion

In the introduction, a holistic approach was identified as a gap, a holistic approach toward technical/nontechnical loss detection. Over a sterile dataset containing only the theft/no theft scenarios or TNT losses/no losses, the known algorithm yields high accuracy, high true positive and low false positive rates. When the dataset includes untagged anomalies in general, the field is such a dataset that it is less likely to work. That was the motivation for TNT loss: (1) detection, (2) classification, and (3) location system. Such a system requires a set of enhancements to known algorithms. It was discussed that such a system would benefit from energy management generation, transmission and distribution several fold. (1) Economically, it is not affordable to waste as much as 15% annually on technical and nontechnical annual losses. (2) Sustainability, fewer losses, less generation, and fewer carbon dioxide emissions indicate less pollution. A problem was identified that an anomaly looks the same for many loss types, not necessarily theft, and not necessarily energy loss, but rather information loss that looks similar to theft, features speaking. This poses a major issue, such as a high actual false positive rate, which is the most serious issue. It is desirable to know with a high confidence level what the loss type is and its location. Identifying the loss type and the loss location along the smart metering data chain to minimize the loss detection handling cost and perform this tagging with not too much preliminary data is desirable. This is the paper’s challenge. Instead of theft/no-theft, loss/no loss—expand the detection to {loss type and loss location}. Loss type detection and loss localization through the electricity load profile only indicate many verified cases of theft, no-theft, and loss of certain types. This information is nonexistent, neither in international datasets nor in utility companies. The redefined problem was shown to be significantly more difficult, and it is impractical based only on energy load profile features to gradually teach the AI to study the loss types. This requires tagged or validated scenario datasets that are too large, and in addition, this will always be one step behind. To obtain that single goal, a collaboration of modules was designed and implemented. These enhancements are not a disconnected cluster of enhancements; they are (i) what it requires to obtain the goal of “loss detection, classification and localization”, (ii) what it requires to obtain theft/no-theft in real field conditions rather than sterile datasets, and (iii) all serve that goal. (1) Several AI modules were implemented, each handling another data channel. This fits the trend of next-generation grid systems to be implemented as multi-agents. (2) The modules were implemented with cross-feedback as “generative cooperative modules”. The AI modules cooperate with (i) data and idea exchange: there was a suggestions generator and discriminator and (ii) simultaneous learning as opposed to separate learning. “Generative cooperative modules” are new to the discipline of “technical and nontechnical loss detection”. (3) The anomaly/normal pooling mechanism was presented and demonstrated over several loss type examples, and eleven types not including theft, which were also presented. That mechanism enables (i) definite loss type identification and (ii) simultaneous validation computation in time of each expert knowledge-based feature. Thus, a much lower training effort than a collaborative all parameters high-order dimensional space was achieved, with a scenarios count and training time of $O\left(NlogN\right)$ instead of $O\left(N^{2}\right)$. “Pooling” usage on feature levels for anomaly type identification is new. (4) A new proposed “robotic process automation” (RPA) design was implemented for loss location, energy losses are located at the premises, and information losses are located along the smart metering data chain and appear similar to energy losses in terms of features. Robotic process automation implemented is a simple yet powerful design routed outside all smart metering software systems and accesses all through the GUI menus with OCR AI technology. The visual recognition is the AI here, and the rest is a flexible software tool. Other RPA implementations were located inside major smart metering software and attempted to access other modules through ports where attempted. For example, a UFT platform was used that does not function properly with software systems, regardless of UFT but due to RPA architectural location. Only outside all “units under test” did the concept work. (5) Human expert knowledge-based features were enhanced from previous work with (i) spectral analysis and (ii) a reactive energy load profile. They were shown to be strongly related to loss/anomaly detection. The reactive energy load profile contains information regarding the load energy loss/anomaly. (iii) The reactive feature was multiplied by all previous features. (iv) The Pearson correlation coefficient (PCC) heatmap was demonstrated to be a 2D meta feature of 256 parameters, an indicator of load profile correlation to loss and to anomaly detection. The three feature ramp features accounted for nine. (6) Shifting from design implementation to the theoretical section, two chapters continued the establishment of the loss/anomaly theory initially presented in the previous work: (i) a “computational complexity” theory of algorithm design provided a closed quantitative estimation formula for the relationship between feature space, (ii) the “required scenarios counted for training” (iii) and mix-up probability between any two loss types, and (iv) the training time was linearly dependent on scenario count. The theory is based on the concept of the “information” contained in the trained algorithm, and that term was quantitated using entropy. A literature survey in the introduction showed that there are similar previous works, but this was the first in computation, at least that is known to our group, for clustering algorithms. Relevancy of the theory to TNT loss (a) explains the role of features space in the TNT loss algorithm’s performance, and (b) alone, the formula enables counting the required scenarios for training and the algorithm accuracy upper bound. It was verified vs. two corner cases of complete mix-up and no mix-up and yields the same results. Most works are empirical: this work enables algorithm computational design. (7) The second theoretical section attempted to lay a mathematical theory of the proposed model of “generative cooperative networks”, which was allocated in image processing. The current architecture was mapped, one variable after the other to that model, which was not simple. Thus, the theory is suitable to describe the proposed TNT loss architecture.

## 5. Conclusions

In this paper, a theoretical model provided a perspective other than architecture presentation to the model statistical ergodic operation and is effective for future research. Four methods were combined to obtain a shortened training time: a) “generative cooperative modules” (GCN) rather than generative adversarial networks (GAN). (1) It is faster to obtain “consensus when the game is cooperative” than to “obtain a discriminator winner when the networks are adversarial”. (2) Training is also simultaneously (!) cooperative, and not only the feedback loops are cooperative. (3) Pooling is time-saving twofold: (i) first, by cutting the high-order dimensional space of dimension *N* constructed of subspaces of dimension *m* into N/m groups, this reduces the computational complexity from $O\left(N^{2}\right)$ to $O\left(NlogN\right)$. (ii) The second is achieved by simultaneously computing each subspace. (4) Data augmentation (DA) cuts the required real training data by producing artificial data. Currently, DA is implemented with simple white Gaussian noise (WGN). DA does not cut computation training time as opposed to items (1)–(3), but it speeds up the time that the system is trained over a verified loss type. Cutting training time enables training after one/few examples of a certain loss type, which is the common scenario and avoids chasing the tail. Real near-time learning of new loss scenarios is needed. The generative cooperative modules take the same design as “generative cooperative networks”, only that AI is initially not a network but rather an expert knowledge-based feature generation module cascaded with a classification algorithm. It was demonstrated that a simple classification and clustering model, such as a logistic classifier, converges quickly with fewer scenarios than other algorithms. It was initially explained that without loss type identification, it is difficult for any TNT loss algorithm to obtain a low “false positive” rate and especially a high true positive rate. Information used by the system. In this work, plenty of typically non-used information that exists in smart metering at the (1) data management (MDM) system, (2) at the billing system (i.e., SAP ERP), and (3) at the workforce management system (WFM). Module data is one for all modules being the same time stamp and vector, and each module uses a different sub-vector of the data. That information is usually not used in TNT loss algorithms, but to resolve this issue, the paper defined two equivalent problems where that information is needed. The first problem was high theft true positives and low theft false positives. The second problem was the identification of TNT {loss type, loss location}. A holistic end-to-end solution attitude was demonstrated utilizing all these data channels: (a) loss events from the meter event log file, finally arriving at the MDM and the data warehouse (DWH), (b) expert user knowledge-based “assertion rules” that were validated manually by TNT loss expert engineers and based on electric parameters measured by the smart meter in addition to energy. These rules were automated by embedding them into the TNT loss system, (c) customer textual data affecting nontechnical loss probability, arriving from the billing system (e.g., SAP ERP), containing at least four information fields related to the probability of loss type: (1) family name, (2) geographic location, (3) implied socioeconomic status, and (4) customer/contract type. All these are now inserted into the TNT loss detection system learning space by using library word2vec, initially developed by Thomas Mikolov, enabling NLP to non-expert users. (c) Pooling vector. Differences from “generative cooperative networks” were discussed: (i) the implemented structures were initially classical machine learning. After stabilization and a sufficiently large dataset, they are replaceable with convolutional neural networks. (ii) The dataset is the same, but the generator is receiving on top of the load profile data. Other data, as described above, are provided only to the generator and not to the discriminator. That is, the same time-stamped data are visualized as a long vector where each module uses other segments. While passing data to the discriminator, this is the load profile mapped 1:1 to the event suggested by the generator. (iii) The training is not performed in advance but on-the-fly. Each new scenario trains the system while it is functioning as a TNT loss, and (iv) there are two time frames: slow and real-time, working simultaneously. In real time, each AI module works independently based on the validated loss type dataset. The slow system is the real GCM. The roles of “generator” and “discriminator” in the TNT loss detection system were explained thoroughly, as well as their training in the forward section and the mathematical section. Referring to the results section, in addition to the demonstration of loss/no-loss, as the previous paper focused on, using new expert knowledge-based features, comparative results using several classification algorithms were tested, and it evolved for the infancy period, for which there were <100 verified thefts, real and synthetic, the simplest logistic regression was the most accurate. For the maturity stage, characterized by theft count > 100, the random forest and decision trees are better. The current paper focuses on (i) experimenting with the suggested economic “pooling” method in the “discriminator” module for loss type identification using pooling. Eleven types of loss were demonstrated. (ii) Loss type localization used the robotic process automation (RPA) module using Eggplant software for this project. There were two counterexamples: load profile information holes and a sudden attenuation of energy consumption. Deterministic data were linked to the “generator”, while AI expert knowledge-based statistics (!) features were linked to the “discriminator”, which gradually learned how to adapt statistical parameters to the anomaly/non-anomaly phenomena scenario. It was noticed, however, starting at the introduction, that the energy load profile from which expert features are generated at the discriminator and the meter event from which a suggestion is raised at the generator are 1:1 mapped to each other. (1) That simplification of the usage of deterministic knowledge first, and AI knowledge second, and turning the deterministic knowledge into “speculative suggestion” is a considerable step that makes the objective feasible. This “deterministic data” approach still required many verified loss-type verified examples. (2) Pooling technology performed the next leap of simplification of TNT loss detection and identification of the job. The results section demonstrates the pooling or decoding for eleven loss types. The bits of the decoded vector were generated from the expert-knowledge features, named alert LEDs. The demonstration was performed using only four events, and it was shown to generate $2^{4}$ loss type signature options. For 14 planned features, this may generate 16,384 options. For the present Day 9 features, this generates 512 features. One of the vector values was 1:1 mapped to a loss type. (iii) The next leap “pooling” was demonstrated to cause the preliminary vision to seem “simpler” to obtain now. The tri-modules (1) generator, (2) discriminator, and (3) “decision-making and probability computation” were shown to be the only cooperative modules, but they are not the only AI modules. (4) There is the “loss location” RPA, implementing OCR visual recognition while using an off-the-shelf product, Eggplant software. (5) An additional AI module was implemented, “word2vec”, converting the textual information into vector information. (iv) In the results section, the multi-agent architecture was demonstrated to be a good architecture for implementation. It identified eleven events out of eleven loss types. With regard to future plans, there the TNT loss detection system will be updated with (a) an operation from the “distribution transformer” CT connected meter, a new AI module. This is a leap from the detection of X10% loss to the detection of ~0.5% loss. A single customer losing 50% was observed at the distribution transformer’s meter as one of a hundred customers. (b) The system will also be enhanced in the future from the distribution grid and to the transmission grid. The latter grid is already covered with meters planned to be used for that task. Next, the paper mapped a theory of “generative cooperative modules” to TNT loss. A “cooperative network” mathematically has an extremely complex dynamic; it is not a zero-sum game reaching Nash’s equilibrium. It is a cooperative learning game reaching consensus, with the Langevin equation describing its dynamics. With a future planned “distribution transformer module”, loss detection will entirely cover the power distribution grids, as deferred from a transmission grid. The system was shown to provide a versatile and accurate solution that is smart metering based on micro-grids. Smart meters are everywhere. It was shown that the TNT loss detection system must be embedded into the smart metering system and collaborate with it via the information. This was named “a holistic approach”. Finally, several algorithmic procedures were proposed to address that. (1) A set of eight consumption expert knowledge-based features for AI learning was demonstrated, and each feature was shown to be an anomaly, non-anomaly detector. (2) With a set of examples, the work has shown that there are anomalies that resemble technical/nontechnical loss detection, such as preventive maintenance and residential theft or true low consumption, for example. The variety of anomalies was shown to pose the main difficulty to any “technical/nontechnical loss detection” system, which causes a high false positive rate. For differentiation between normal and abnormal, the features already show a definite difference at each of the features—level, rather than in high-order dimensional spaces, and finally by accurate figures using classification algorithms. (3) Reviewing the system flow: the system was shown to function best if the technical/nontechnical loss detection lifetime was split into three stages: (3.1) infancy, up to fifteen verified frauds in the local dataset, the simplest algorithm, a logistic classifier (LR), performed with the best accuracy because it converges quickly based on a few verified frauds. In that stage, the secondary robotic process automation (RPA) algorithm was shown to differentiate between information loss located along the smart metering data chain and energy loss located at the meter location and to locate the anomaly along the smart metering data chain. Where does failure exist? The subsystem was shown to cover eleven loss types. (3.2) The maturity stage is determined by the availability of approximately 100 verified frauds in the local dataset. The same applies to the other loss types. In that stage, nonlinear classifier random forest (RF) and decision tree (DT) classifiers are better performers than a logistic classifier (LR). In addition, anomalies detected by the RPA sub-algorithm are tagged and added to the local dataset. Now, the technical/nontechnical loss detection system gradually learns to identify this variety of anomalies by using the tagged examples and the wealth of approximately 512 expert feature parameters and pooling. The RPA submodule co-works with the TNT loss detection algorithm to identify loss locations and reduce false positive scenario counts. (3.3) “Steady-state” is characterized by 1000 verified frauds in the local dataset. In this stage, the AI can separate various anomalies through expert features, and deep learning may be implemented for classification. (4) It has also been shown by research, again, a holistic approach, that when all expert features are executed, then it is one-to-one mapping of loss type using the pooling technology; therefore, all eight features are needed. In addition, it was shown that identification and classification are required prior to dispatching a team to the field, all the other loss types. The conclusion of the research is that to operate a technical/nontechnical loss detection system in real practice and a nonsterile theft/no-theft tagged dataset, a variety of implemented AI modules are needed. (5) It was demonstrated that it is a procedure of conditional probability computation of loss types. This was shown to be critical to reducing the false positive rate. Deterministic in nature of the information, yet speculative due to missing functioning sensors, information arrives from the smart metering system. Then, it enters the learning space. (6) It was demonstrated that the reactive energy load profile channel contributes additional information to technical and nontechnical loss determination and to a lesser extent than an active energy load profile. (7) It was shown that the Pearson correlation heatmap with respect to fraud may reduce 512 variables to a 2D image for loss type identification. Hence, a correlation heatmap was shown to have threefold benefits: (a) for turning loss type identification into 2D visual object recognition, (b) as a feature by itself correlating to TNT loss identification and loss type, and (c) to clearly indicate that reactive is less correlated to fraud than active on average. A multi-agent approach was demonstrated to be scalable, with each module taking a different processing role, and the method of co-work was generative cooperative modules. The decision was achieved through consensus. Most importantly, the impossible tasks of loss type identification, classification and location are made possible by this work and appear to be much more attainable. Finally, the disadvantages of the system are mentioned. (1) It is more complex than a single AI module/network processing than an energy load profile. Thus, it may be answered that the system goals are more complex than simple theft detection, and obtaining low false positives is also a goal. (2) It takes more time to train due to two time-cycle systems: fast, similarly to most TNT loss systems and slow, which is new to the proposed system. (3) It consumes more scenarios. There is a lack of “loss type” validated scenarios. It requires an “energy loss data augmentation module”. The existing worldwide electricity theft detection datasets may be insufficient for loss-type system training. (4) The robotic process automation platform is a somewhat expensive technology. This is a powerful automation technology that relieves the requirement to automate through communication ports between modules and enables communication through GUIs; that technology enables loss location.

## Figures and Tables

**Table 1:** Comparative results of various algorithms with expert knowledge preprocessors versus other works.

			Fraud			Non-Fraud		
Model	Accuracy Macro, Weighted	Precision	F1-Score	Recall	Accuracy	Precision	F1-Score	Recall
Proposed SVM + HDS^2^	0.81	0.81	0.5	0.33	0.81	0.62	0.77	1
Proposed Ridge + HDS	0.81 0.8	1	0.55	0.33	0.81 0.8	0.81	0.77	1
Proposed KNN + HDS	0.88	1	0.800	0.67	0.88	0.77	0.67	1
Proposed RF + HDS	0.92 0.91	1	0.88	0.78	0.92 0.91	0.83	0.91	1
Proposed DT + HDS	0.95 0.95	1	0.94	0.89	0.95 0.95	0.91	0.95	1
Proposed LR + HDS	1 1	1	1	1	1 1	1	1	1
Wide & deep CNN [24]	0.9503	0.9503	0.9093	--	--	--	--	--
Work								
SVM w/o preprocess	0.772	0.765	0.863	--	--	--	--	--
LR without preprocess	0.676	0.645	0.937	--	--	--	--	--
CNN	0.812	0.805	0.845	--	--	--	--	--
RUSBoost	0.869	0.85	0.871	--	--	--	--	--
Work with [59] preprocessing and supervised learning	0.95	0.93	0.937	--	--	--	--	--

**Table 2:** Listing down the faults detected by the TNT loss system.

			Fraud			Non-Fraud		
No.	Loss Type	Initially, Raised by Module	Currently Identified by Module	Was Verified Yes/No	Unique Signature Yes/No	Reason, Details	Final System Decision	Comment
1	Magnetic tampering	Module 2: events	Module 2: events Module 1: AI	yes	no	It is a false event by sensor, there is no real nontechnical loss	For the specific model type tag this is not a fault	It is not a tampering loss, it is a meter false alert
2	Disconnected phase	Module 2 events	Module 2: events Module 1: AI	yes	yes	Three mechanisms alert this now: (i) Meter event, (ii) expert knowledge rule: voltage ≠0 while current = 0, (iii) AI signature	Tag this as a true TNT Loss	A true event 14 m out of 50,000 identified
3	Reversed-phase	Module 2 events: assertion rule	Module 2: events Module 1: AI	yes		Three mechanisms alert this now: (i) Meter event, (ii) expert knowledge rule: active export ≠0, (iii) AI signature and (iv) must not be PV	Tag this as a true TNT loss	A true event two meters out of 50,000 identified
4	Repeated meter restart	Module 2 events	Module 2: events Module 1: AI	yes	yes	Due to hardware failure causing repeated restarts, energy stored in temporary registers is lost, and energy not measured during restart time is lost—metrological damage	Tag this as a true TNT loss	A true event five meters out of 50,000 identified
5	Meter turn-off/not measured	Module 2 events, assertion rule	Module 2: events Module 1: AI	yes	yes	Due to hardware failure the meter stops measuring	Tag this as a true TNT loss	A true event 14 m out of 50,000 identified
6	Meter abruptly low consumption	Module 1 AI, Module 2 events—assertion rule	Module 1: AI	yes	yes	Firmware defect: during daily midnight clock synch with SNTP an internal multiplication factor is reduced to close to zero but not zero	Tag this as a true TNT loss	A true event 5 m out of 50,000 identified
7	Holes/gaps in load profile	Module 1: AI	Module 2 events—assertion rule	yes	yes	Due to architecture flaw—lack of handshaking at load profile transfer from MDM to DWH^1^, 50% of the data gaps were generated. Insertion of a handshaking system TALEND resolved the problem completely	Tag this as a true TNT loss	A true event occurring at 100% of meters out of 50,000 identified
8	A random value of 20,000–2,000,000 kWh is exerted at one of three phases as export. From there, meter counts are precise	Module 2 events, assertion rule	Module 2: events Module 1: AI	yes	yes	Root cause analyzed this occurs at the factory. It is missed at the acceptance test by pulse counting and detected by electronic meter reading	Tag this as a true TNT loss	A true event occurring at 0.05% of meters out of 50,000 identified
9	Memory access fault events	Module 2 events, assertion rule	Module 2: events Module 1: AI	yes	yes	Might cause energy loss due to miss storage of energy	Tag this as a true TNT Loss	A true event occurring at 0.05% of meters out of 50,000 identified
10	Meter abruptly low consumption at a distribution transformer and data concentrator CT connected meter	Module 1 AI, Module 2 events—assertion rule	Module 1: AI	yes	yes	At distribution transformer level this fault causes energy balance mismatch—energy, which is more severe than a single faulty meter	Tag this as a true TNT Loss	A true event occurring at 0.05% of meters out of 50,000 identified
11	Signal quality	Module 2 events	Module 2 events	no	no	Power quality events. This is nota fault	Tag this as a false TNT Loss	A false event occurring at = ~0.05% of meters out of 50,000 identified

## Data Availability

The MATLAB and Python executable codes used in this research are available for academic purposes only by contacting the corresponding author (D.S.). The new dataset used in this study is freely available at https://github.com/grid-dev-group/papers (accessed on 30 August 2020).

## Abbreviations

RPA	Robotic process automation
GCN	Generative cooperative networks
GCM	Generative cooperative (AI) modules (classical not deep neural networks)
GAN	Generative adversarial network
TNT losses	Technical nontechnical losses
NEW_FL	Tagging a group of new untagged failures
Word2Vec	an NLP library converting from text to vector
SAP ERP	Systems applications and products in data processing, enterprise resource planning
a universal billing system	
EUT	Equipment under test
RDP	Remote desktop protocol
OCR	Optical character recognition
MDM	Meter data management system
WFM	Workforce management system
NLP	Natural language processing
HES	Head end system
PLC	Power line carrier communication method
PCA	Principal component analysis
DWH	Data warehouse
TALEND	“Data integrity and governance” system
